# QTL Mapping of a Novel Genomic Region Associated with High Out-Crossing Rate Derived from *Oryza longistaminata* and Development of New CMS Lines in Rice, *O. sativa* L.

**DOI:** 10.1186/s12284-021-00521-9

**Published:** 2021-09-16

**Authors:** G. D. Prahalada, Balram Marathi, Ricky Vinarao, Sung-Ryul Kim, Reynaldo Diocton, Joie Ramos, Kshirod K. Jena

**Affiliations:** 1 Novel Gene Resources Laboratory, Strategic Innovation Platform, International Rice Research Institute, DAPO Box 7777, Metro Manila, Philippines; 2 PJ Telangana State Agricultural University, Hyderabad, Telangana 500030 India; 3 School of Biotechnology, KIIT University, Bhubaneswar, Odisha 751024 India

**Keywords:** Long-exserted stigma, QTL mapping, Out-crossing rate, *O. longistaminata*, *O. sativa*, Hybrid rice

## Abstract

High seed cost due to poor seed yield severely limits the adoption of hybrid rice by farmers. Increasing the out-crossing rate is one of the key strategies to increase hybrid seed production. Out-crossing rate is highly influenced by the size of female floral traits, which capture pollen grains from male donor plants. In the current study, we identified 14 QTLs derived from the perennial wild rice *Oryza longistaminata* by composite interval mapping for five key floral traits: stigma length (five), style length (three), stigma breadth (two), stigma area (one), and pistil length (three). QTL analysis and correlation studies revealed that these stigma traits were positively correlated and pleiotropic to the stigma length trait. We selected the major-effect QTL *qSTGL8.0* conferring long stigma phenotype for further fine mapping and marker-assisted selection. The *qSTGL8.0* (~ 3.9 Mb) was fine mapped using newly developed internal markers and was narrowed down to ~ 2.9 Mb size (RM7356–RM256 markers). Further, the flanking markers were validated in a segregating population and in progenies from different genetic backgrounds. The markers PA08-03 and PA08-18 showed the highest co-segregation with the stigma traits. The *qSTGL8.0* was introgressed into two cytoplasmic male sterile (CMS) lines, IR58025A and IR68897A, by foreground, background, and trait selection approaches. The *qSTGL8.0* introgression lines in CMS backgrounds showed a significantly higher seed setting rate (2.5–3.0-fold) than the original CMS lines in test crosses with their corresponding maintainer lines. The newly identified QTLs especially *qSTGL8.0*, will be quite useful for increasing out-crossing rate and this will contribute to increase seed production and decrease seed cost.

**Supplementary Information:**

The online version contains supplementary material available at 10.1186/s12284-021-00521-9.

## Background

Rice is the staple food for more than half of the world's population and it provides more than 20% of the daily caloric intake of more than 3.5 billion people (Ray et al. 2013). It is estimated that an additional 116 million tons of rice will be needed by 2035 to feed the world's growing population (http://ricepedia.org/rice-as-food/the-global-staple-rice-consumers). In contrast, Green Revolution technologies that had paved the way for increasing annual yield (3.00%) have exhausted further productivity gains, with annual yield gains falling to 1.25% since 1990 (FAO 1996). Furthermore, rice yields in most South and Southeast Asian countries appear to be approaching a plateau.

Beginning in the early 1970s, significant research efforts have gone into developing hybrid rice, which is shown to have a yield advantage up to 20% higher than that of conventional Green Revolution high-yielding varieties (Peng et al. 1998, 2003; Katsura et al. 2007; Bueno and Lafarge 2009). It was during the early 1970s that Chinese researchers discovered a wild-abortive cytoplasmic male sterile (WA-CMS) rice plant on Hainan Island that led to the development of hybrid rice breeding in China, where hybrid rice has been grown commercially since 1976, surpassing 6.0 t ha^−1^ in yield. Hybrid rice has been commercialized on a large scale particularly in China and it covers more than 50.0% of the total rice-planted area and accounts for about two-thirds of the national production. However, transferring Chinese hybrid rice technology to other Asian countries has proven difficult.

Development of a male sterile (MS) line is one of the prerequisites for the production of hybrid seeds. Initially, the development of hybrid rice varieties used the CMS genetic male sterility (CGMS) system or three-line breeding system as it was convenient and efficient. This system uses a CMS line (also called an A line); a maintainer (B) line (an isogenic line of A except for the cytoplasm and hence being fertile) that, when crossed with the A line, produces MS offspring; and a restorer (R) line that, when crossed with the A line, produces fertile hybrid seeds. Another system of male sterility called two-line was developed during the mid-1990s based on the type of gene(s) conferring male sterility (Cheng et al. 2007). The male sterility resulting from the interaction of nuclear genes with environmental conditions such as photoperiod and temperature was named photosensitive genetic male sterility (PGMS) and thermo-sensitive genetic male sterility (TGMS), respectively (Li et al. 2009a, b). Although PGMS and TGMS have several advantages, the dependence on temperature and day length makes implementation tricky and imposes temporal and geographic limits on hybrid seed production (Li et al. 2009a, b). Notably, whatever the type of male sterility system used for the development of hybrid rice, the seed yield of the hybrids in seed production depends mainly on the out-crossing rate. Hence, as early as the mid-1980s, increasing the out-crossing rate of MS lines became a major target in hybrid rice breeding (Virmani and Athwal 1973; Taillebois 1983; Zhou et al. 2009).

Cultivated rice is predominantly self-fertilizing due to the morphology of its flower, shorter anthers and stigma, and pollen released shortly after the florets open (Oka 1988). Out-crossing rates in cultivated rice varieties have diminished along with changes in the morphology of rice flowers during the process of domestication, giving out-crossing rate of 0.01% (Sahadevan and Namboodiri 1963; Li et al. 2009b). The low rate of out-crossing causes poor hybrid seed production (seed set of 5–20%), resulting in high costs for purchasing hybrid rice seeds. These two factors have been cited as major constraints to the wider and faster adoption of hybrid rice varieties by rice farmers (Xie 2009). Hence, it is imperative to develop CMS lines with improved out-crossing rate that can diminish the cost of hybrid seed production.

Out-crossing rate in the female parent is mainly influenced by floral traits such as stigma size (length and breadth), length of style, stigma exsertion, and angle and duration of glume opening, whereas, in the male parent, it is influenced by anther size, number of pollen grains per anther, filament length, and duration of spikelet blooming (Virmani 1994). Importantly, among these traits, stigma length and stigma exsertion possess high correlation toward increasing out-crossing rate in the seed parent (Marathi and Jena 2015).

Wild species, being reservoirs of essential traits, are used in crop improvement for transferring high-value traits (Ramos et al. 2016). The extent of out-crossing is predicted to be higher in wild rice than in cultivated rice, indicating preference for open pollination similar to the progenitor *Oryza perennis*, which was partially allogamous (Oka and Morishima 1967). Among the wild rice species, the out-crossing rate varied from 3.2 to 70.0%. Certain accessions of wild rice, *O. longistaminata* (OL) and *O. rufipogon* (formerly *O. perennis*), have shown out-crossing rates of up to 100% (Sakai and Narise 1959; Oka and Morishima 1967). Interestingly, among the AA genome wild species, OL possesses desirable floral characteristics, specifically stigma and style length, that enhance out-crossing rate (Marathi and Jena 2015). Hence, OL can be used for the transfer of long-exserted stigma and other out-crossing rate-influencing floral traits into maintainer lines toward the development of new CMS lines that can enhance out-crossing rate.

The genetics of floral traits (stigma length, stigma exsertion, and style length) as studied by several researchers revealed that these traits were influenced by polygenes with additive and non-additive gene action (Virmani and Athwal 1974; Zhou et al. 2017). Although several QTLs influencing floral traits were identified from both cultivated and wild rice sources, none of them were introgressed into CMS line backgrounds to validate the genetic effect of the identified QTLs and further to evaluate out-crossing rate. Hence, in our study, we have (1) identified several QTLs for the important floral traits through linkage analysis using a BC_2_F_2_ mapping population derived from IR64 and OL as recipient and donor parents, respectively; (2) fine-mapped the major-effect QTL *qSTGL8.0* to obtain the best marker-trait association; (3) introgressed the trait into the background of two CMS lines by marker-assisted backcrossing (MABC) following foreground, background, and phenotypic selection approaches; and (4) developed novel CMS lines with higher out-crossing rate and stable male sterility.

## Results

### Phenotypic Characterization of Parental Lines and Development of Mapping Populations for Stigma Traits

**Fig. 1:**
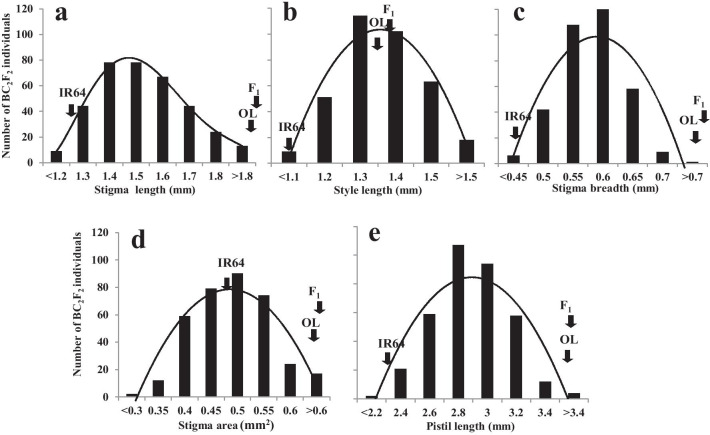
Frequency distribution of floral traits **a** stigma length, **b** style length, **c** stigma breadth, **d** stigma area, and pistil length in an IR64 × OL(IRGC110404)-derived BC_2_F_2_ mapping population. Arrow indicates the phenotypic value of IR64, F_1_, and OL plants for the respective floral trait

In our previous study on pistil traits of wild rice species (Marathi et al. 2015), we suggested that OL possessed an ideal female organ structure (a long and exserted stigma phenotype) for increasing seed setting rate in hybrid seed production. Hence, in this study, we used an OL (IRGC110404) to develop mapping populations through crosses with *O. sativa* (IR64) and furthermore to identify the genetic loci controlling stigma traits. First, five pistil traits (stigma length, style length, stigma breadth, stigma area, and pistil length) were phenotyped from parents as described in material and methods (Additional file 4: Fig. S1). As expected, the OL exhibited higher values for all five traits than those of IR64, especially for stigma length (OL: 2.39 mm, IR64: 1.31 mm) (Table 1). F_1_ plants were obtained through wide hybridization between IR64 and OL (IRGC110404). All five stigma phenotypes of F_1_ plants were the same as those of OL (*α* = 0.05) (Table 1). Further, F_1_ plants generated 37 BC_1_F_1_ and 37 BC_2_F_1_ plants after backcrossing to the recurrent parent and the BC_2_F_1_ plants were self-pollinated to produce 357 BC_2_F_2_ plants. A total of 3,570 florets were collected and dissected for phenotyping of the key floral traits among the 357 BC_2_F_2_ segregating plants. The mean performance and the range of the trait values obtained from the mapping populations indicated segregation toward the cultivated parent for all the traits. The frequency distribution scores showed bell-shaped curves for each of the traits studied and partially skewed toward cultivated rice lines (Fig. 1). The mean values of each trait were used for the linkage analysis for locating the loci influencing these key floral traits.

**Table 1:** Phenotypic characterization of parental lines and F_1_ (IR64 × OL) for stigma traits

Genotypes	Stigma length (mm)	Style length (mm)	Stigma breadth (mm)	Stigma area (mm^2^)	Pistil length (mm)
IR58025A	1.46	1.01	0.50	0.44	2.47
IR58025B	1.44	1.07	0.47	0.51	2.51
IR64	1.31	1.05	0.46	0.53	2.35
IR68897A	1.43	0.87	0.72	0.67	2.29
IR68897B	1.51	1.13	0.63	0.61	2.64
OL (IRGC110404)	2.39	1.29	0.97	1.13	3.56
F_1_ (IR64 × OL)	2.46	1.31	0.92	1.15	4.07
SE (±)	0.15	0.02	0.02	0.04	0.05

A correlation study was carried out to assess the correlations among the floral traits like spikelets with exserted stigma (%), stigma length (mm), pistil length (mm), and internal angle of stigma lobes. The highest significant correlation coefficient was noticed between stigma length and pistil length (0.95) at the 0.01 level of significance, followed by that between stigma length and stigma exsertion (0.86) and between stigma exsertion and pistil length (0.78). The positive significant correlation coefficients at a lower level of significance indicated that the traits stigma length, stigma exsertion, and pistil length were positively correlated. Hence, selection of anyone of these traits positively influences the selection of other traits (Additional file 1: Table S1).

### Linkage Map Construction and Localization of Genomic Regions Associated with Stigma Traits

**Fig. 2:**
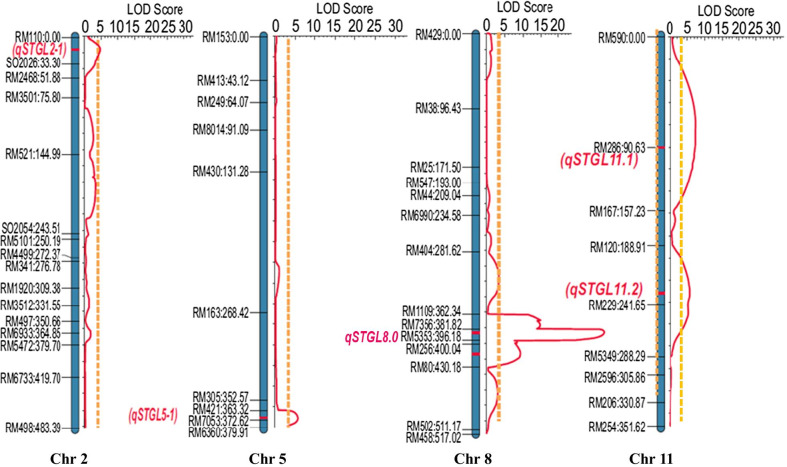
Diagram showing the QTLs detected for stigma length (*qSTGL2-1, qSTGL5-1, qSTGL8.0, qSTGL11-1,* and *qSTGL11-2*) by composite interval mapping using 357 BC_2_F_2_ segregants derived from IR64 × OL

A mapping population consisting of 357 BC_2_F_2_ plants was genotyped by 164 polymorphic SSR and STS markers and a saturated linkage map was constructed. The genotypic and phenotypic data of pistil traits were used to map the genomic regions conferring each floral trait. Composite interval mapping identified 14 QTLs in total on different chromosomes; 5, 3, 2, 1, and 3 QTLs for stigma length, style length, stigma breadth, stigma area, and pistil length, respectively (Table 2). Among the QTLs detected, the major QTL (*qSTGL8.0*) bordered by RM1109 and RM80markers on the long arm of chromosome 8 showed the highest phenotypic variance of 35.40%, with 42.50 LOD for stigma length (Table 2; Fig. 2). For style length, three QTLs (*qSTYL1-1, qSTYL5-2*, and *qSTYL8-1*) were detected on chromosomes 1, 5, and 8, respectively. Among these QTLs, *qSTYL8-1* showed the highest phenotypic variance (17.11%) and was identified between marker intervalsRM404and RM1109 on chromosome 8.8 For stigma breadth, two QTLs (*qSTGB1-1* and *qSTGB3-1*) were detected and QTL *qSTGB1-1* showed the highest phenotypic variance (21.14%), with an LOD of 14.71. However, only one QTL (*qSTGA8-1*) was detected on the long arm of chromosome 8 for stigma area, with an LOD of 8.52 and phenotypic variance of 3.12%. Furthermore, for pistil length, three genomic regions (*qPSTL1-1, qPSTL1-3*, and *qPSTL11-1*) were identified and one of the QTLs, *qPSTL11-1* on chromosome 11 with an LOD value of 5.63, explained 26.96% of the phenotypic variance (Additional file 6: Fig. S3).

**Table 2:** List of floral trait QTLs detected in IR64 × OL BC_2_F_2_mapping population by composite interval mapping at 0.01 level of confidence and 1000 permutations

Floral traits	Number of QTLs detected	QTL	Chromosome	Flanking marker (left)	Flanking marker (right)	LOD	Additive	Dominance	R^2^
Stigma length	5	*qSTGL2-1*	2	RM110	S02026	4.60	− 0.02	0.20	8.61
		*qSTGL5-1*	5	RM421	RM7653	5.60	0.07	− 0.07	3.51
		*qSTGL8.0*	8	RM1109	RM80	42.50	− 0.10	0.10	35.40
		*qSTGL11-1*	11	RM590	RM286	7.40	0.00	0.10	4.30
		*qSTGL11-2*	11	RM120	RM229	5.70	− 0.10	-0.10	6.80
Style length	3	*qSTYL1-1*	1	RM319	RM3640	9.97	0.10	0.00	15.10
		*qSTYL5-2*	5	RM7653	RM6360	6.12	− 0.08	0.01	10.21
		*qSTYL8-1*	8	RM404	RM1109	4.58	0.06	-0.06	17.11
Stigma breadth	2	*qSTGB1-1*	1	RM403	RM319	14.71	− 0.04	0.00	21.14
		*qSTGB3-1*	3	RM3525	RM520	9.77	0.04	0.01	9.09
Stigma area	1	*qSTGA8-1*	8	RM404	RM7356	8.52	0.04	0.03	3.12
Pistil length	3	*qPSTL1-1*	1	RM3604	RM3746	8.06	− 0.13	-0.02	8.03
		*qPSTL1-3*	1	RM3640	RM8134	8.59	0.15	0.05	9.00
		*qPSTL11-1*	11	RM5997	RM254	5.63	0.25	-0.12	26.96

**Fig. 3:**
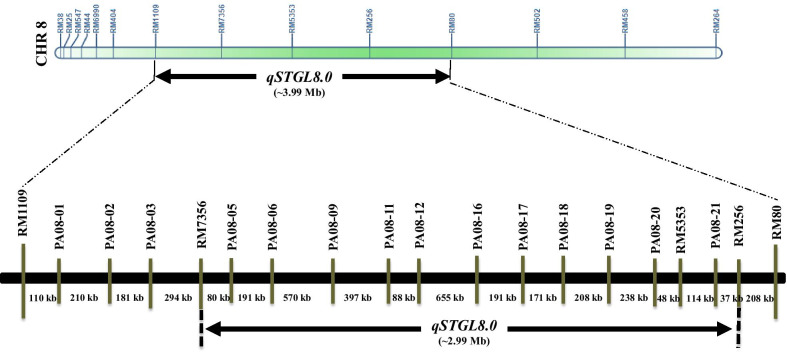
The *qSTGL8.0* locus with flanking markers*. qSTGL8.0* detected between SSR markers RM7356 and RM80 (~ 3.99 Mb) using 357 BC_2_F_2_segregants derived from IR64 × OL; was narrowed down to 2.99 Mb by using newly designed InDel markers

To dissect the major-effect QTL *qSTGL8.0* that was mapped between markers RM1109and RM80 corresponding to 3.99 Mb size of the reference genome (IRGSP1.0), 21 InDel markers were newly designed based on a sequence comparison between OL and the reference genome within the flanking marker positions (Additional file 2: Table S2). Of the 21 InDel markers, 14 showed polymorphism between IR64 and OL. These 14 markers were used to genotype a 357 BC_2_F_2_ mapping population. We carried out additional linkage analysis using the new genotypic data and previously collected phenotypic data and narrowed down the locus to 2.99 Mb size bordered by RM7356 and RM256 (Fig. 3). Further, the tightly linked markers for the long-stigma phenotype were identified through a marker validation experiment using 135 BC_2_F_3_ plants derived from the two BC_2_F_2_ plants (BC_2_F_2_-8 and BC_2_F_2_-51) with the same set of 14 markers. In the segregating BC_2_F_3_ plants, long stigma phenotype was dependent on the genotype of *qSTGL8.0*, regardless of the genotypes of the four minor *qSTGL*, suggesting that *qSTGL8.0* is the major QTL and the genetic effect of the minor QTLs are not clear. The marker PA08-18 was highly co-segregating with the phenotype and less than 3% recombination was found between the marker and trait (Additional file 7: Fig. S4), suggesting that the causal gene for long stigma istightly linked to the PA08-18 marker. Hence, these markers were used for the introgression of the locus *qSTGL8.0* into maintainers and CMS lines (Fig. 3).

### Evaluation of the Genetic Effect of *qSTGL8.0* in Commercial Maintainer Lines

**Fig. 4:**
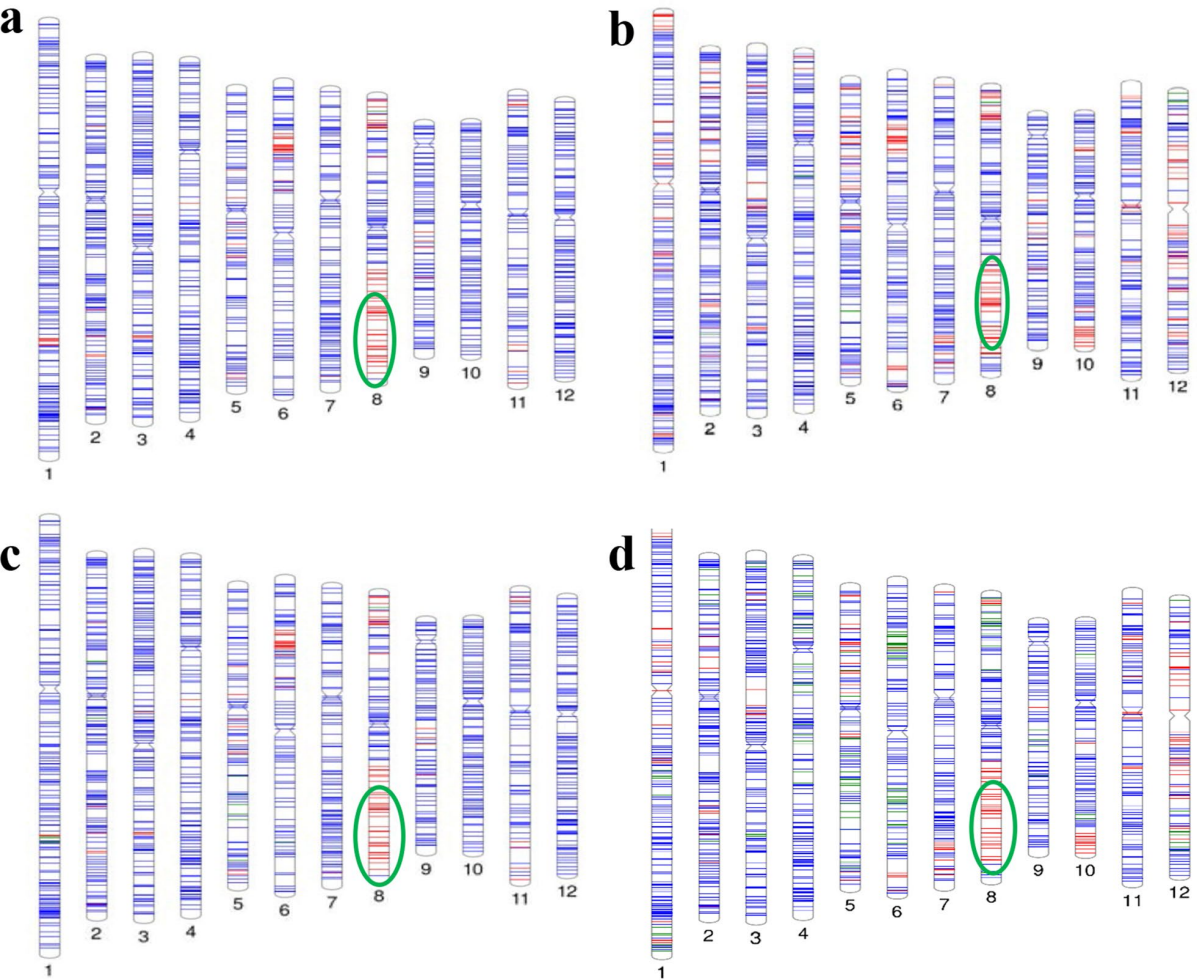
Graphical genotype map of the improved maintainer lines **a** 91B-42 and **b** 107B-12 and the improved CMS lines **c** 91A-18 and **d** 107A-35. This map was constructed based on the genotypic data of the high-density polymorphic SNP markers (Infinium 7 K SNP chip). Numbers below each chromosome indicate the respective chromosome number. *Blue, red,* and *green* lines indicate the recurrent parent, donor parent, and heterozygous SNP alleles, respectively. The *qSTGL8.0* segment introgression is highlighted by a green circle

To evaluate the genetic effect of *qSTGL8.0* in different genetic backgrounds, the QTL was introgressed into two commercial maintainer (B) and CMS (A) lines, IR58025B/A and IR68897B/A, which had shorter stigma (1.43‒1.51 mm) and smaller size in other pistil traits than those of OL (Table 1). Line IR58025B was crossed with the OL (IRGC110404) and line IR68897B was crossed with another OL accession (IRGC92664) also exhibiting long stigma, and *qSTGL8.0* was transferred into each maintainer background by the MABC method (Additional file 8: Fig. S5). Briefly, the F_1_ plants were backcrossed to the corresponding B line and genotyping was conducted using 10 markers (PA08-03, PA08-05, PA08-06, PA08-09, PA08-11, PA08-12, PA08-16, PA08-17, PA08-18, and PA08-19) covering the *qSTGL8.0* locus with 103 (IR58025B × OL-IRGC110404) and 98 (IR68897B × OL-IRGC92664) BC_1_F_1_ plants. Marker-trait association analysis revealed that the BC_1_F_1_ plants possessing OL alleles at *qSTGL8.0* had long-exserted stigma phenotype while the plants of the maintainer lines had short-stigma phenotype in both backgrounds. The BC_1_F_1_ plants possessing the *qSTGL8.0-*OL allele were backcrossed and produced 29 and 14 BC_2_F_1_ seeds in IR58025B and IR68897B backgrounds, respectively. Further, the lines were advanced to the BC_2_F_3_ generation based on the genotypic data. A total of 158 BC_2_F_3_ plants having the homozygous OL allele at the *qSTGL8.0* locus (59 in IR58025B and 99 in IR68897B backgrounds) were obtained and they showed significantly long-exserted stigma vis-à-vis the original recurrent parents as well as the BC_2_F_3_ segregants possessing the homozygous recurrent allele of *qSTGL8.0* (Additional file 9: Fig. S6). These results indicated that the genetic effect of the *qSTGL8.0*-OL allele was clearly seen in two different maintainer backgrounds in addition to IR64, and both the *qSTGL8.0* alleles derived from two OL accessions (IRGC110404 and IRGC92664) are functional and increase stigma length in *O. sativa*. To examine the genome recovery status of the introgressed lines, background genotyping of the selected BC_2_F_4_ plants was carried out using high-density SNP markers (Illumina 7 K SNP chip). The percent recovery of the recurrent parent genome was computed among the selected BC_2_F_4_ plants. The BC_2_F_4_ plants 91B-42 (IR58025B background) and 107B-12 (IR68897B background) showed maximum recovery of the recurrent parent genomes, 92.43% and 90.35%, respectively, carrying the homozygous OL alleles at the *qSTGL8.0* locus (Additional file 3: Table S3; Fig. 4). The stigma length of the lines 91B-42 and 107B-12 was 2.43 mm and 2.08 mm, respectively, while it was 1.30 mm for IR58025B and 1.36 mm for IR68897B (Additional file 3: Table S3). Hence, these improved maintainer lines with maximum recurrent parent genome recovery were used as donor maintainer lines for the transfer of *qSTGL8.0* to their corresponding CMS lines (Additional file 8: Fig. S5).

### Transfer of *qSTGL8.0* from the Improved Maintainer Lines to the Corresponding CMS Lines

The improved B lines were test-crossed with sterile CMS lines IR58025A and IR68897A to transfer the *qSTGL8.0* locus, which could enhance out-crossing rate. Hence, during the2014 dry season, 134 test crosses were made between the improved B lines and their corresponding CMS lines (IR58025A and IR68897A) to transfer *qSTGL8.0* into a parental CMS line background. Based on pollen sterility and stigma traits, 19 F_1_ plants were selected and backcrossed with the respective improved IR58025B (Line 91B-42) and IR68897B (Line 107B-12) lines, andBC_1_F_1_ seeds were produced. A total of 101 BC_1_F_1_ plants underwent foreground selection for the *qSTGL8.0* locus by using the flanking markers. The BC_1_F_1_ plants with long-exserted stigma possessing the homozygous OL allele at the *qSTGL8.0* locus and being completely pollen sterile were selected and backcrossed again to develop BC_2_F_1_ plants and then BC_3_F_1_ plants (Additional file 8: Fig. S5b). Background analysis of 91A-18 plants selected from BC_3_F_1_ progenies in the background of IR58025A showed as high as 92.21% recurrent parent genome recovery. Similarly, the improved CMS line 107A-35 derived from IR68897A showed as high as 94.48% recurrent parent genome recovery (Additional file 3: Table S3; Fig. 4). The improved CMS lines 91A-18 and 107A-35 showed stigma lengths of 2.18 mm and 2.61 mm, respectively, which were significantly longer than those of their background parents, IR58025A and IR68897A (Additional file 3: Table S3). These results suggested that the transfer of a single major QTL, *qSTGL8.0*, among the 14 QTLs detected in our study significantly increased stigma length in two different CMS lines and therefore the genetic effect of *qSTGL8.0* was validated in all the backgrounds tested.

### Phenotypic Evaluation of Parental and Improved CMS Lines

**Fig. 5:**
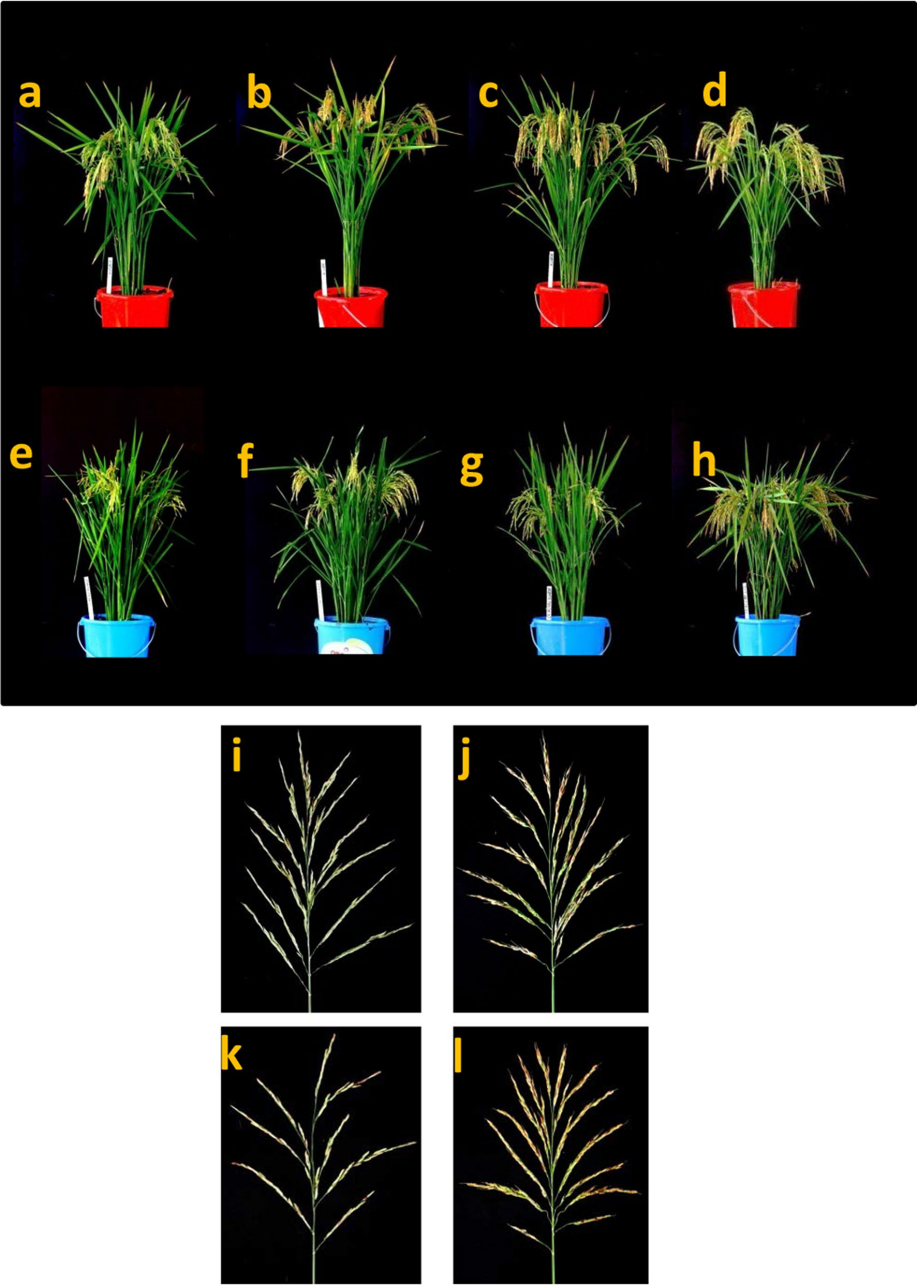
Photographs showing the plant architecture of **a** IR58025B, **b** 91B-42, **c** IR68897B, and **d** 107B-12 maintainer lines and **e** IR58025A, **f** 91A-18, **g** IR68897A, **h** and 107A-35A CMS lines and seed setting in the panicle of **i** IR58025A, **j** 91A-18, **k** IR68897A, and **l** 107A-35

The improved CMS lines 91A-18 and 107A-35 along with the original parental CMS lines (IR58025A and IR68897A) were evaluated for agro-morphological traits and seed setting rate. The improved CMS lines showed similar trait performances for most of the traits studied (Table 3; Fig. 5). For the traits such as plant height and panicle number, the recurrent parent and improved CMS lines showed a similar performance. The CMS line IR58025A (82.67 cm) and its improved line 91A-18 (83.33 cm) were significantly taller than the other CMS line, IR68897A (74.67 cm), and its improved line, 107A-35 (72.88 cm). On the contrary, with 28.67 and 24.01 mean number of panicles, IR68897A and 107A-35 possess significantly more tillers than IR58025A (18.01) and its improved line, 91A-18 (16.10). However, plot yield and seed setting rate were significantly higher in the improved CMS lines than in both the recurrent CMS lines. All the CMS lines were pollinated with the respective B lines. Plot yield was 2076.11 kg ha^−1^ and 2172.72 kg ha^−1^ for the recurrent parents, IR58025A and IR68897, respectively, while it was 2431.92 kg ha^−1^ and 2832.72 kg ha^−1^ for 91A-18 and 107A-35, respectively. Similarly, the seed setting rate of the recurrent parents was 22.72% for IR58025A and 31.86% for IR68897A, whereas it was 69.36% for 91A-18 and 77.88% for 107A-35. This result clearly showed that there was an enhanced out-crossing rate of at least 2.50 times (245%) that of the recurrent parent IR58025A, whereas it was 3.05 times that of IR68897A. Nevertheless, the pollen sterility of all the CMS lines was higher than 99.90% consistently, indicating stable expression of male sterility across several seasons. These results suggested that a long-exserted stigma phenotype induced by *qSTGL8.0*-OL alleles significantly improved plot yield and seed setting rate in CMS backgrounds.

**Table 3:** Performance of parental and improved CMS lines for yield and yield-associated traits

Genotypes	PH (cm)	PN	PL (cm)	PY (kg ha^−1^)	OC (%)	S (%)
IR58025A	82.67 ± 1.12^b^	18.01 ± 1.76^a^	24.50 ± 0.32^b^	2076.11 ± 7.38^b^	22.72 ± 3.93^b^	99.99
91A-18	83.33 ± 1.13^b^	16.10 ± 1.62^a^	26.10 ± 0.30^a^	2431.92 ± 7.06^a^	69.36 ± 3.60^a^	99.99
IR68897A	74.67 ± 1.05^ab^	28.67 ± 1.67^b^	21.33 ± 0.36^c^	2172.22 ± 7.71^b^	31.86 ± 3.72^b^	99.98
107A-35	72.88 ± 1.06^a^	24.01 ± 1.79^b^	28.09 ± 0.44^a^	2832.72 ± 7.75^ac^	77.88 ± 5.34^a^	99.99

^abc^are LSD significant values at 0.05 level of significance

*PH* plant height, *PN* panicle number, *PL* panicle length, *PY* plot yield, *OC* outcrossing rate, *S* pollen sterility

Values are means ± SE

### Assessment of Stigma Receptivity of Parental and Improved CMS Lines

**Fig. 6:**
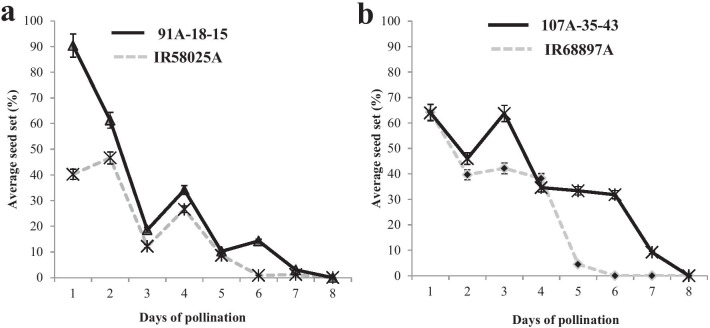
Graphical representation of stigma receptivity trend of **a** IR58025A and 91A-18-15 and **b** IR68897A and 107A-35-43

As stigma receptivity is the ability of the stigma to support viable and compatible pollen and is also one of the contributors for out-crossing rate, an experiment was conducted to determine the duration of stigma receptivity of the improved CMS lines possessing *qSTGL8.0* and their original CMS lines, IR58025A and IR68897A. First, the stigma length of improved CMS lines and their recurrent parental CMS lines was characterized. Then, the same sets of lines were evaluated for studying the duration of stigma receptivity. As expected, the stigma length of improved CMS lines 91A-18–15 (2.62 mm) and 107A-35–43 (2.25 mm) was significantly higher than that of their background CMS lines, IR58025A (1.37 mm) and IR68897A (1.48 mm) (Table 4). Further, to study the duration of stigma receptivity, out-crossing rate (seed setting percentage) was considered as the measure of the duration of stigma receptivity from day one until the day when the lowest or no seed setting was computed. On the first day, the out-crossing rate of the improved A line (91A-18–15) was 90.35% whereas it was 40.24% in the background parent, IR58025A. The out-crossing rate for IR58025A was nil on the sixth day, whereas it was still 14.28% in the improved CMS line (91A-18–15). Similarly, for IR68897A, the out-crossing rate was nil on the sixth day, whereas it was 31.82% for improved CMS line 107A-35–43 (Fig. 6). These results indicated that stigma receptivity gradually decreased from the spikelet opening day in both recurrent and improved CMS lines; however, stigma receptivity was slightly longer in the improved CMS lines than in the original CMS lines.

**Table 4:** Stigma trait characters of the parental and improved CMS lines

Genotypes	Stigma length (mm)	Style length (mm)	Stigma breadth (mm)	Stigma area (mm^2^)	Pistil length (mm)
IR58025A	1.37^b^	1.04^c^	0.46^d^	0.38^c^	2.60^b^
91A-18-15	2.62^a^	1.22^a^	0.96^a^	0.99^a^	3.84^a^
IR68897A	1.48^b^	0.86^b^	0.70^c^	0.58^bc^	2.34^b^
107A-35-43	2.25^a^	1.50^a^	1.02^a^	1.19^a^	3.75^a^
IR64	1.31^c^	1.05^c^	0.46^d^	0.53^bc^	2.36^b^
OL	2.39^a^	1.29^a^	0.97^a^	1.13^a^	3.68^a^
SE( ±)	0.11	0.06	0.03	0.07	0.06

^abcd^are LSD significant values at 0.05 level of significance

## Discussion

The production of rice, being the staple food in most Asian countries, has to be increased through the exploitation of heterosis breeding to meet the food security challenges of the twenty-first century (Yuan and Peng 2005; Fan et al. 2015). However, low hybrid seed production due to the poor out-crossing rate of the female parent is one of the major constraints that not only limits hybrid rice development but also its adoption in rice farmers’ fields in Asian countries (9.40% in Vietnam, 6.80% in Bangladesh, 4.30% in the Philippines, 3.20% in India, and 0.50% in Indonesia) (Barclay 2010). Hence, increasing the out-crossing rate represents priority research for hybrid seed production (Taillebois 1983; Taillebois et al. 2017). Despite many efforts for genetic improvement of out-crossing rate in hybrid seed production, there is no clear advancement yet, such as improvement of stigma traits in hybrid parental lines and higher out-crossing rate. Here, we identified a handful of QTLs governing stigma traits from a wild rice of African origin, *O. longistaminata*. Furthermore, we showed the strong possibility to increase out-crossing rate through the development of long-exserted stigma maintainer/CMS lines possessing the newly identified QTL, *qSTGL8.0*, and its evaluation.

Out-crossing rate is highly influenced by several floral traits such as stigma length, stigma exsertion, style length, stigma area, stigma breadth, and pistil length (Virmani and Athwal 1973; Zhou et al. 2009; Marathi et al. 2015). It was revealed that these floral traits were positively correlated with a higher out-crossing rate (Kato and Namai 1987a, b; Miyata et al. 2007; Liu et al. 2015; Taillebois et al. 2017). Especially, seed setting rate is highly influenced by stigma exsertion in male sterile plants (Parmar et al. 1980; Hoff and De La Torre 1981) and long stigma is regarded as the major factor for high stigma exsertion (Parmar et al. 1980). In our study, we also obtained similar results by trait-phenotypic characterization and correlation studies that indicated significantly positive correlation, particularly between stigma length and stigma exsertion, with higher rates of out-crossing (Additional file 1: Table S1). Hence, our study focused on the development of novel CMS lines with enhanced out-crossing rate of at least 60% by introgression of the long-stigma QTL, *qSTGL8.0*, from the wild species, OL (Table 3; Additional file 8: Fig. S5). These new CMS lines could play a key role in decreasing the seed production cost of both parental (A × B) and hybrid (A × R) lines, and thus increasing the potential of hybrid rice adoption.

Initially, in order to study the extent of genetic variability among the parental lines, phenotypic characterization was performed for all the female floral traits. Among all the test lines studied, OL showed longer stigma, style, and pistil; broader stigma; and larger stigma area for harnessing sufficient pollen grains to achieve higher out-crossing rate. Hence, OL was used for the identification of genomic regions influencing these floral traits. Interestingly, the F_1_ progenies obtained from the cross of IR64 and OL also showed a similar phenotypic performance as the donor parent, OL. This result suggests that the genetic loci controlling the key floral traits are dominant, especially for stigma length (Table 1).The frequency distribution and genetic analysis of the traits obtained from the measurements of stigma length, style length, stigma breadth, stigma area, and pistil length of the mapping population showed a normal distribution pattern with continuous variation, unlike the classical Mendelian bimodal distribution. This finding suggests that these floral traits are controlled by polygenes with cumulative and additive effects, and are influenced by environmental factors. Our result is in agreement with the findings of other researchers (Yan et al. 2009; Kato and Namai 1987a, b; Liu et al. 2015).

Several genetic factors, including QTLs and genes for the several female floral traits, have been identified from *O. sativa* and *O. rufipogon*. At least 26 QTLs conferring stigma length were detected using eight different mapping populations on all chromosomes of rice, except chromosome 11. For style length, 11 QTLs were reported on six chromosomes in five mapping populations (Uga et al. 2003; 2010; Yan et al. 2009), whereas 26 QTLs were identified for stigma exsertion rate (Li et al. 2001; Uga et al. 2003; Yu et al. 2006; Miyata et al. 2007; Yan et al. 2009; Hu et al. 2009). For stigma breadth (stigma width), 17 QTLs were identified on chromosomes 1 to 7, 9, and 12 (Li et al. 2001; Uga et al. 2003; 2010). Among these QTLs detected, *qSTB-12* was found to be a major QTL showing as high as 30.50% phenotypic variance in a recombinant inbred line population derived from Pei-kuh and W1944, which is an introgressed line from the wild species *O. rufipogon* (Uga et al. 2003). In our study, we have detected QTLs conferring different floral traits using a set of a 357 BC_2_F_2_ mapping population derived from a cross between IR64 and OL. A total of five QTLs associated with stigma length are detected on chromosomes 2, 5, 8, and 11, whereas three QTLs (*qSTYL1-1, qSTYL5-2*, and *qSTYL8-1*) for style length are located on chromosomes 1, 5, and 8, respectively. Similarly, we detected the QTLs*qSTGB-1* and *qSTGB3-1*for stigma breadth; *qSTGA8-1* for stigma area; and *qPSTL1-1*, *qPSTL1-3,* and *qPSTL11-1* for pistil length. We identified novel QTLs *qSTGL2-1, qSTGL5-1, qSTGL11-1*, and *qSTGL11-2*for stigma length; *qSTYL1-1*, *qSTYL5-2*, and *qSTYL8-1* for style length; *qSTGB1-1* and *qSTGB3-1* for stigma breadth; *qSTGA8-1* for stigma area; and *qPSTL1-1*, *qPSTL1-3*, and *qPSTL11-1* for pistil length (Marathi and Jena 2015; Dang et al. 2016). Although Uga et al. (2010) reported the QTLs *qSTYL1-1* and *qSTGB1-1* conferring style length and stigma breadth, respectively, on chromosome 1, these QTLs are located far away from the QTLs we identified in our study. Interestingly, the floral trait QTLs *qSTGL8.0* for stigma length, *qSTYL8-1* for style length, and *qSTGA8-1* for stigma area are identified on chromosome 8 and are overlapping (Fig. 2; Additional file 6: Fig. S3), notably on the long arm region, suggesting that the common genomic region on chromosome 8 may regulate three stigma traits above. In addition, we also observed strong positive co-relation between stigma length and style length (Additional file 1: Table S1). Therefore, there is possibility that the major QTL *qSTGL8.0* is associated with style length and stigma area as well as stigma length. Hence, the *qSTGL8.0* genomic region has been introgressed in the background of the parental lines IR64, IR58025A/B, and IR68897A/B, which accelerates the simultaneous improvement of the lines for different floral traits.

The extent of marker-trait association determines the success of MAS. Fine mapping decreases the chance of recombination, which otherwise causes poor marker-trait association (Xu and Crouch 2008). Hence, in our study, we initially localized the *qSTGL8.0* locus in an approximate size of 3.99 Mb and dissected this further to a 2.99 Mb genomic region flanking the markers RM7356 and RM256. In the fine-mapped genomic region, no recombination occurred between the markers and locus as evidenced by the marker validation experiment we conducted using 135 BC_2_F_3_ (IR64 × OL) and103 (IR58025B × OL) and 98 (IR68897B × OL) BC_1_F_1_ plants. We used the same markers flanking these regions for the introgression of the traits/genes through MAS in the background of elite lines.

During the introgression of *qSTGL8.0* into the CMS background lines IR58025A and IR68897A using the improved maintainer lines, foreground selection was carried out using the highly co-segregating flanking markers PA08-03 and PA08-18. The positive plants were further backcrossed and advanced to generate BC_3_F_1_ progenies. These plants underwent background analysis to select the plants with maximum genome recovery of the recurrent parent possessing the target locus. The genome recovery was as high as 92.21%, which was noticed in the improved CMS line (91A-18) in the background of IR58025A, whereas 94.48% genome recovery was observed for improved CMS line (107A-35) derived from background line IR68897A (Additional file 3: Table S3; Fig. 4). Efforts are ongoing to advance these lines through MABC and select the plants with the highest genome recovery of the recurrent parent genome. Additionally, the same improved maintainer lines and their CMS lines were evaluated to study the stigma length and duration of stigma receptivity. As expected, these improved CMS lines have shown significantly long-exserted stigma compared to their background parents and longer duration of stigma receptivity (Table 4). The stigma receptivity of these lines was at least for 7 days compared with that of their parental lines, which showed 2 to 4 days of stigma receptivity, similar to that of most of the cultivated *indica* rice varieties (Xu and Shen 1988). Eventually, the improved CMS lines with extended duration of stigma receptivity harvest pollen grains up to 7 days after the spikelet opening, which increased the number of seeds set and hence the out-crossing rate (Fig. 6).

The two CMS lines (IR58025A and IR68897A) used in our study are the most popular CMS lines being used in hybrid rice breeding programs worldwide, especially in South and Southeast Asia, respectively, mainly because of their agro-morphological characteristics, combining ability, and grain quality parameters. Hence, phenotypic selection was carried out to select improved CMS lines confirmed with the *qSTGL8.0* locus in the respective background CMS lines, showing maximum recurrent parent genome recovery and long-exserted stigma with desirable agro-morphological characters. Our results showed that, for most of the agronomic characters, including plant height and tiller number, the improved CMS lines 91A-18 and 107A-35 had a performance similar to that of their background parents (Table 3; Fig. 5). One of the critical observations from the agronomic trait evaluation experiment was the enhanced out-crossing rate of the improved CMS lines vis-à-vis that of both background parents. This is one of the remarkable achievements of our study aimed at increasing hybrid seed production. This could be achieved through the identification of genomic regions conferring the floral trait QTLs and introgression of the stigma traits, especially stigma length, in the background CMS lines that had only about a 30% out-crossing rate (Table 3). However, in every step of CMS line development, selection of pollen sterile plants was performed to ensure complete sterility of the improved CMS lines toward stable CMS line development. Notably, these improved CMS lines not only show an enhanced out-crossing rate because of *qSTGL8.0* introgression but also express similar agro-morphological characters as their recurrent parents, for which those recurrent parents are being used extensively in hybrid rice breeding programs. The CMS lines with long-exserted stigma that can harvest a surplus amount of pollen grains also show a long duration of stigma receptivity, which will definitely increase the out-crossing rate. In our study, we have demonstrated the identification and introgression of novel QTLs conferring floral traits into two popular CMS lines. We strongly believe that the newly identified QTLs associated with stigma traits derived from OL as well as the improved maintainer lines and their corresponding improved CMS lines possessing *qSTGL8.0* will be valuable genetic resources for increasing the out-crossing rate for both three-line and two-line hybrid seed production systems, and this will eventually help to decrease hybrid seed cost and accelerate hybrid rice adoption, especially in Asian countries, which will ensure global food security.

## Methods

### Plant Materials

The wild rice species OL (two accessions: IRGC110404 and IRGC92664) belonging to the AA genome complex possessing desirable floral characters including stigma length for the improvement of hybrid rice was used as a donor parent (Marathi and Jena 2015). The high-yielding elite *indica* rice cultivar IR64 was used as the recipient variety for the development of mapping populations and identification of genomic regions associated with stigma traits. For the validation of the newly detected QTLs in different genotypic backgrounds, the detected QTLs were introgressed into two popular commercial *indica* hybrid parental lines, IR58025B/A and IR68897B/A, by the MABC method.

### Development of Mapping Population

F_1_ seeds were produced from the cross between OL (IRGC110404) and cultivar IR64. The true F_1_ plants were selected by morphological and molecular marker analysis and they were used as female parents for backcrossing with IR64 to produce 267 BC_1_F_1_ seeds. Based on their phenotypic similarity with IR64 and stigma length trait similar to OL, 37 BC_1_F_1_ plants underwent another round of backcross and 220 BC_2_F_1_ plants were produced. Finally, 357 BC_2_F_2_ plants were generated from the 37 BC_2_F_1_ plants showing long-exserted stigma phenotype. Further, these plants were genotyped and phenotyped for molecular mapping of the floral traits. Initial crosses and backcrosses were made in the screen house and the BC_2_F_2_ plants were grown in an experimental field at the International Rice Research Institute (IRRI) (14.20° N and 121.20° E), Philippines. The schematic presentation of population development is shown in Additional file 5: Fig. S2.

### Phenotyping of Spikelet Traits

A total of five major female spikelet traits (stigma length, style length, stigma breadth, stigma area, and pistil length) that might be associated with high out-crossing rate were measured from dissected spikelets. Stigma length is the total length of brushy and non-brushy regions of the pistil, stigma area is the length and breadth of the stigma, style length is the length of filaments of the bifid stigma, and pistil length is the total length of the style, ovary, and stigma (Additional file 4: Fig. S1). However, as observed from previous studies, there was no significant difference between the parental lines for the length of non-brushy area of the stigma (Marathi and Jena 2015; Marathi et al. 2015); therefore, the length of brushy area in the stigma was considered as stigma length in our study.

For the phenotypic characterization of floral traits, spikelets were collected at the anthesis stage (spikelet opening time in the morning) from the top, middle, and bottom parts of the panicles and were put into vials containing 70% ethanol to avoid rupturing of the stigma and its parts. The collected spikelets were dissected to isolate female parts under a stereo microscope (Leica MS5) and the specimens were observed under an Olympus® CX23 stereomicroscope to capture images. The images were analyzed using Image-Pro Plus version 7.0 software to measure the length and area of the female organ. The obtained measurement values were documented in a Microsoft spreadsheet for further statistical analysis. For each genotype, 10 spikelets per plant were dissected from five plants of uniform parental lines and ten pistils from individual plants of segregating populations and measured to obtain phenotypic values.

A correlation study was conducted to analyze the relatedness of the stigma traits stigma length, stigma exsertion, pistil length, and angle between the lobes. The parental lines IR64 and OL were used to estimate the correlation coefficients for all the stigma traits. The highest positive significant correlation coefficients indicate strong linkage of stigma traits.

### Screening of DNA Markers, Genotyping of Mapping Populations, and Linkage Analysis

A set of 922 simple sequence repeat (SSR) and sequence tagged site (STS) markers distributed over the 12 rice chromosomes was surveyed for parental polymorphism between IR64 and OL (IRGC110404). As a result, 164 markers were found polymorphic and used for genotyping of the mapping populations. Genomic DNA was isolated from the fresh leaf tissues of individual BC_2_F_2_plants and their parental lines using the modified CTAB DNA extraction method as described by Kim et al. (2011). PCR was carried out with 164 polymorphic markers following normal PCR conditions (35 cycles of 95 °C for 25 s, 55 °C for 25 s, and 72°Cfor 35 s). Amplification products were separated by either 3.0% agarose gel electrophoresis or 8.0% non-denaturing polyacrylamide gel electrophoresis (PAGE). The genotypes for each marker were scored as “A” (homozygous for IR64), “H” (heterozygote), and “B” (homozygous for OL). The Kosambi mapping function (Kosambi 1944) was used for the estimation of recombination fraction and MAPMAKER/EXP 3.0 (Lincoln et al. 1992) was used to construct the linkage map from the 164 markers spanning the 12 rice chromosomes. Furthermore, the genomic regions conferring different floral traits were located using the mean values of phenotypic data (10 spikelets/plant) and genotypic data from the 164 markers. QTL IciMapping software version 4.0 (Meng et al. 2015) with 1000 permutations at 0.01 significance LOD threshold was used for the linkage analysis and the retrieved results were validated in WinQTL cartographer version 2.50 (Wang et al. 2012) considering the same threshold parameters.

### Development of the *qSTGL8.0* Flanking Markers

The sequence alignment data between OL and *O. sativa* subsp. *japonica* var. Nipponbare (IRGSP 1.0) provided by the Gramene database (http://www.gramene.org/) were used for the development of InDel-type markers*.* The newly designed markers were tested for polymorphism between IR64 and OL, and the polymorphic markers were used for genotyping the same mapping population, BC_2_F_2_ (357), which was used for the primary mapping.

### Marker-Assisted Selection for Validation of *qSTGL8.0*

A total of 135 BC_2_F_3_ plants derived from the two BC_2_F_2_ plants that were used for QTL analysis were used for the marker validation. Marker-trait association analysis between genotypic data from the flanking markers and phenotypic data (stigma length) was conducted and the percent co-segregation of markers was computed. The genetic effect of the major QTL *qSTGL8.0* conferring long-exserted stigma was validated using the markers underlying the locus. The major-effect QTL was transferred by MABC into two different *indica* hybrid parental backgrounds (IR58025B and IR68897B). The genotype and phenotype of 103 and 98 BC_1_F_1_ plants derived from IR58025B × OL (IRGC110404) and IR68897B × OL (IRGC92664) crosses, respectively, were used for the QTL validation. The markers with maximum percent co-segregation from three different background genomes were considered as tightly linked markers and used for introgression of the *qSTGL8.0* locus into three different genome backgrounds: IR64, IR58025B/A, and IR68897B/A.

Flanking markers of the major- and minor-effect QTLs conferring long-exserted stigma with maximum significant co-segregation were used for foreground selection for the development of improved maintainer and CMS lines possessing long-exserted stigma. The foreground selection was performed in the BC_1_F_1_ generation and in further backcross and selfing generations for selecting genotypes with the target locus. The positive plants were further advanced through foreground and phenotypic selection from BC_1_F_1_to BC_2_F_4_ generations for developing improved maintainer lines. Similarly, for developing improved CMS lines, foreground and phenotypic selection were practiced to generate BC_3_F_1_ plants. The genetic background recovery of the BC_2_F_4_ progenies of the improved maintainer lines and BC_3_F_1_ progenies of improved CMS lines derived from the positive plants possessing the target locus was determined using a high-density SNP marker genotyping platform, Illumina Infinium 7 K SNP chip (Thomson et al. 2017). The genotyping was carried out at the genotyping service laboratory of IRRI (http://gsl.irri.org/). SNP genotyping data were retrieved in the HapMap format. The retrieved raw SNPs were processed and a graphical genotype map was generated following the methodology described by Prahalada et al. (2017). The breeding scheme for the development of improved CMS lines is presented in Additional file 8: Fig. S5.

Phenotypic evaluation and selection were also carried out among the BC_3_F_1_ progenies of A lines that were found to carry major-effect QTLs influencing stigma traits and having high recurrent parent genome recovery to select for desirable yield and yield-associated traits. For the evaluation of the newly developed CMS lines, the agro-morphological characters (including plant height, panicle number per plant, and panicle length) were measured. In addition, spikelet fertility (out-crossing rate) and plot yield were tested by crossing with the corresponding B line. The evaluation of genotypes was conducted in both the wet and dry seasons following a randomized complete block design (RCBD) with two replications during 2015, 2016, and 2017. A total of 25 plants and five panicles from each plant were used for the data recording.

### Pollen Fertility Studies

For assessing pollen fertility, three spikelets were collected from the bottom, middle, and top positions of the main panicle from five plants in each entry and fixed in 70% ethyl alcohol. Pollen grains were squeezed out from the anthers on a clean glass slide, stained with iodine-potassium iodide solution (100 mg I_2_, 1 g KI, 100 mL H_2_O), and examined under a light microscope (Olympus BX53). The pollen grains were considered to be fertile if they were plump, round, and deeply stained, whereas they were considered sterile if they were shrunken, unstained, and irregular in shape. The total numbers of fertile and sterile pollens were counted for a minimum of 300 pollen grains. Percent pollen fertility was calculated in percentage as the ratio of the total number of fertile pollens to the total number of pollens. During the development of improved CMS lines using the newly identified QTL, the plants of CMS lines with 100% pollen sterility were backcrossed and advanced further for the development of stable CMS lines.

### Assessment of Stigma Receptivity

The duration of stigma receptivity was determined using 10flowering plants of the parental lines IR58025A and IR68897A along with their improved lines showing a long-exserted stigma phenotype. A total of three flowering panicles per plant were selected, the opening spikelets at the day were maintained, and the other spikelets (already opened and not yet opened) were removed from each panicle. Tips of spikelet from the remaining florets were cut and anthers were removed so that stigmas were exposed, then the panicles were bagged. From the next day onward, the plants were pollinated by their corresponding B line for 10 days with 1-day intervals. Seeds were harvested 25 days after pollination. In total, five plants per genotype on each pollination day were analyzed and stigma receptivity was presented as percentage (total number of seeds set/total number of spikelets × 100). The number of days was counted for the duration of stigma receptivity until the day when the out-crossing rate became the lowest or zero.

### Statistical Analysis

Analysis of variance (ANOVA) was used to separate out the total variance of all the stigma traits and other agro-morphological traits. It was carried out using average values obtained from five panicles of 25 plants during the two seasons of three years. The experimental RCBD was employed to study the genetic variability of parental lines IR64, OL, IR58025B, and IR68897B and their introgression lines. Two tailed *t*-test and Fisher’s least significant difference (LSD) test at *α* = 0.05 and/or 0.01 level of significance were used to compare the means of the test entries and infer the significant difference between the cultivars under study. Composite and multiple interval mapping that are based on strong statistical power and maximum likelihood (multiple regression analysis) were employed for the molecular mapping of floral traits influencing out-crossing rate*.* The mode of genetic segregation of floral traits was analyzed using the statistical test, chi-square goodness-of-fit, and frequency distribution of traits.

## Conclusion

A major QTL (*qSTGL8.0*) associated with long exserted stigma trait from *O. longistaminata* was detected by QTL mapping. Upon validation, the *qSTGL8.0* has been transferred into two popular CMS lines, IR58025A and IR68897A through genomics assisted introgression. The improved CMS lines showed enhanced out-crossing rate without being compromising basic traits. We believe that the detected QTL and improved CMS lines can contribute to reduce the cost of hybrid seed production and hence, increased area under hybrid rice cultivation which ultimately helps in food security.

## Supplementary Information

**Additional file 1**: Table S1. Correlation coefficients of the key floral traits.

**Additional file 2**: Table S2. List of newly designed polymorphic InDel markers with their sequences and product sizes.

**Additional file 3**: Table S3. Background genotyping analysis of the improved maintainer and CMS lines using high-resolution Infinium 7K SNP chip.

**Additional file 4**: Figure S1. Schematic diagram showing the different parts of the typical OL female reproductive organ (pistil). The letters a, b, c, d, e, and f denote non-brushy parts of the stigma, brushy parts of the stigma, stigma, style, ovary, and pistil, respectively.

**Additional file 5**: Figure S2. Breeding scheme depicting the development of a mapping population derived from IR64 × OL (IRGC110404).

**Additional file 6**: Figure S3. QTLs detected from 357 BC2F2 genotypes derived from an IR64 × OL cross conferring (a & b) style length, (c) stigma breadth, (d) stigma area, and (e) pistil length using 164 SSR and STS markers.

**Additional file 7**: Figure S4. Agarose (3%) gel image showing the BC2F3 co-segregation pattern of the new PA08-18 InDel marker predicted to link to qSTGL8.0. Marker alleles were scored as A for IR64 alleles, B for OL alleles, and H for heterozygous alleles of IR64 and OL for genotype score assessment. Phenotype below the genotype scores indicates length of the phenotype of the respective BC2F3 individuals.

**Additional file 8**: Figure S5. Schematic diagram showing the breeding scheme of an IR68897B ×OL(IRGC92664) cross for the development of improved maintainer (a) and CMS lines (b) with long-exserted stigma and higher out-crossing rate than recurrent parents IR68897B and IR68897A, respectively. The same breeding scheme was used for an IR58025B ×OL (IRGC110404) cross to develop long-exserted stigma lines in IR58025B and IR58025A backgrounds.

**Additional file 9**: Figure S6. Phenotypes of panicle, floret, and stigma. (a) IR68897B. (b) OL (IRGC110404). (c) The improved IR58025B possessing qSTGL8.0 (IRGC110404). (d) The improved IR68897B possessing qSTGL8.0 (IRGC92664). Red arrows point to the exserted stigma in each genotype.

## Data Availability

All datasets supporting the results and conclusions of this manuscript are included in the article and supplementary files. Materials generated in the study are maintained in Novel Gene Resource Group of Strategic Innovation Platform at the International Rice Research Institute, Los Baños, Philippines.

## References

[CR1] Barclay A (2010). Hybridizing the world. Rice Today.

[CR2] Bueno CS, Lafarge T (2009). Higher crop performance of rice hybrids than elite inbreds in the tropics: 1. Hybrids accumulate more biomass during each phenological phase. Field Crops Res.

[CR3] Cheng SH, Zhuang JY, Fan YY, Du JH, Cao LY (2007). Progress in research and development on hybrid rice: a super-domesticate in China. Ann Bot.

[CR4] Dang X, Liu E, Liang Y, Liu Q, Breria CM, Hong D (2016). QTL detection and elite alleles mining for stigma traits in *Oryza sativa* by association mapping. Front Plant Sci.

[CR5] Fan F, Li N, Wang J, Liu X, Liu J, Zhu Y, Li S (2015). Molecular marker-directed development of a novel cytoplasmic male sterile line in rice. Mol Breed.

[CR6] FAO (1996) Food for all. World Food Summit, Rome. http://www.fao.org/3/x0262e/x0262e06.html

[CR7] Hoff BJ, De La Torre M (1981) Stigma exsertion in rice and its effect on the seed set of male-sterile plants. In: 73rd annual progress report of the Rice Experiment Station, Crowley, Louisiana, pp 240‒243

[CR8] Hu S, Zhou Y, Zhang L, Zhu X, Wang Z, Li L, Luo L, Zhou Q (2009). QTL analysis of floral traits of rice (*Oryza sativa* L) under well-watered and drought stress conditions. Genes Genom.

[CR9] Kato H, Namai H (1987). Floral characteristics and environmental factors for increasing natural out-crossing rate for F1 hybrid seed production of rice *Oryza sativa* L. Jpn J Breed.

[CR10] Kato H, Namai H (1987). Inter-varietal variations of floral characteristics with special reference to F1 seed production in Japonica rice (*Oryza sativa* L.). Jpn J Breed.

[CR11] Katsura K, Maeda S, Horie T, Shiraiwa T (2007). Analysis of yield attributes and crop physiological traits of Liangyoupeijiu, a hybrid rice recently bred in China. Field Crops Res.

[CR12] Kim SR, Jeon JS, An G (2011). Development of an efficient inverse PCR method for isolating gene tags from T-DNA insertional mutants in rice. Methods Mol Biol.

[CR13] Kosambi DD (1944). The estimation of map distance from recombination values. Ann Eugenics.

[CR14] Li C, Sun CQ, Mu P, Chen L, Wang XK (2001). QTL analysis of anther length and ratio of stigma exsertion, two key traits of classification for cultivated rice (*Oryza sativa* L.) and common wild rice (*O. rufipogon* Griff.). Acta Genet Sin.

[CR15] Li J, Xin Y, Yuan LP (2009a) Hybrid rice technology development. Ensuring China’s food security, IFPRI

[CR42] Li X, Xiao J, Xie F, Yuan L (2009). Modified single cross for hybrid rice breeding. Accelerating hybrid rice development.

[CR16] Lincoln S, Daly M, Lander E (1992) Constructing genetic maps with Mapmaker/exp 3.0. In: Whitehead institute technical report, Cambridge, MA, USA

[CR17] Liu QM, Qin JC, Li TW, Liu EB, Fan DJ, Edzesi WM, Liu J, Jiang J, Liu X, Xiao L, Liu L, Hon D (2015). Fine mapping and candidate gene analysis of qSTL3, a stigma length-conditioning locus in rice (*Oryza sativa* L.). PLoS ONE.

[CR18] Marathi B, Jena KK (2015). Floral traits to enhance out-crossing for higher hybrid seed production in rice: present status and future prospects. Euphytica.

[CR19] Marathi B, Ramos J, Hechanova SL, Oane RH, Jena KK (2015). SNP genotyping and characterization of pistil traits revealing a distinct phylogenetic relationship among the species of *Oryza*. Euphytica.

[CR20] Meng L, Li H, Zhang L, Wang J (2015). QTL IciMapping: integrated software for genetic linkage map construction and quantitative trait locus mapping in biparental populations. Crop J.

[CR21] Miyata M, Yamamoto T, Komori T, Nitta N (2007). Marker assisted selection and evaluation of the QTL for stigma exsertion under japonica rice genetic background. Theor Appl Genet.

[CR22] Oka HI (1988). Origin of cultivated rice.

[CR23] Oka HI, Morishima H (1967). Variations in the breeding systems of a wild rice, *Oryza perennis*. Evolution.

[CR24] Parmar KS, Swaminathan MS, Siddiq EA (1980). Variation in reproductive organs of rice with reference to male incompatibility index. Indian J Genet Plant Breed.

[CR25] Peng S, Yang J, Garcia FV, Laza MRC, Siddiq EA, Muralidharan K, Virmani SS (1998). Physiology-based crop management for yield maximization of hybrid rice. Advances in hybrid rice technology.

[CR26] Peng S, Yang J, Laza MRC, Sanico AL, Visperas RM, Son TT, Virmani SS, Mao CX, Hardy B (2003). Physiological bases of heterosis and crop management strategies for hybrid rice in the tropics. Hybrid rice for food security, poverty alleviation, and environmental protection. Proceedings of the 4th international symposium on hybrid rice.

[CR27] Prahalada GD, Shivakumar N, Lohithaswa HC, Sidde Gowda DK, Ramkumar G, Kim SR, Ramachandra C, Hittalmani S, Mohapatra T, Jena KK (2017). Identification and fine mapping of a new gene, *BPH31* conferring resistance to brown planthopper biotype 4 of India to improve rice, *Oryza Sativa* l. Rice.

[CR28] Ramos JM, Furuta T, Uehara TK, Chihiro N, Angeles-Shim RB, Shim J, Brar DS, Ashikari M, Jena KK (2016). Development of chromosome segment substitution lines (CSSLs) of *Oryza longistaminata* A. Chev. & Ro¨hrin the background of the elite japonica rice cultivar, Taichung 65 and their evaluation for yield traits. Euphytica.

[CR29] Ray DK, Mueller ND, West PC, Foley JA (2013). Yield trends are insufficient to double global crop production by 2050. PLoS ONE.

[CR30] Sahadevan PC, Namboodiri KMN (1963). Natural crossing in rice. Proc Indian Acad Sci.

[CR31] Sakai KI, Narise T (1959). Studies on the breeding behavior of a wild rice (*Oryza rufipogon* and *Oryza perennis*). Inst Genet Jpn.

[CR32] Taillebois J (1983). New prospects for the production of F_1_ hybrid seed: transfer of allogamous characters of *O. longistaminata* A. Chev to *O. Sativa* l. Agron Trop.

[CR33] Taillebois J, Dosmann J, Cronemberger H, Paredes H, Cao T, Neves P, Ahmadi N (2017). Breeding for out-crossing ability in rice, to enhance seed production for hybrid rice cropping. J Rice Res.

[CR34] Thomson MJ, Singh N, Dwiyanti MS, Wang DR, Wright MH, Perez FA, De Clerck G, Chin JH, Malitic-Layaoen GA, Juanillas VM, Dilla-Ermita CJ, Mauleon R, Kretzschmar T, McCouch SR (2017). Large-scale deployment of a rice 6 K SNP array for genetics and breeding applications. Rice.

[CR35] Uga Y, Fukuta Y, Cai HW, Iwata H, Ohsawa R, Morishima H, Fujimura T (2003). Mapping QTLs influencing rice floral morphology using recombinant inbred lines derived from a cross between *Oryza sativa* L. and *O. rufipogon* Griff. Theor Appl Genet.

[CR36] Uga Y, Siangliw M, Nagamine T, Ohsawa R, Fujimura T, Fukuta Y (2010). Comparative mapping of QTLs determining glume, pistil and stamen sizes in cultivated rice (*Oryza sativa* L.). Plant Breed.

[CR37] Virmani SS, Virmani SS (1994). Prospects of hybrid rice in the tropics and subtropics. Hybrid rice technology: new developments and future prospects.

[CR38] Virmani SS, Athwal DS (1973). Genetic variability in floral characteristics influencing out-crossing in *Oryza sativa* L. Crop Sci.

[CR39] Virmani SS, Athwal DS (1974). Inheritance of floral characteristics influencing out-crossing in rice. Crop Sci.

[CR40] Wang S, Basten CJ, Zeng ZB (2012) Windows QTL cartographer 2.5. Department of Statistics, North Carolina State University, Raleigh

[CR41] Xie F, Xie F, Hardy B (2009). Priorities of IRRI hybrid rice breeding. Accelerating hybrid rice development.

[CR43] Xu Y, Shen Z (1988). Receptivity of exserted stigma. Intl Rice Res Newsl.

[CR44] Xu Y, Crouch JH (2008). Marker-assisted selection in plant breeding: from publications to practice. Crop Sci.

[CR45] Yan WG, Li Y, Agrama HA, Luo DG, Gao FY, Lu XJ, Ren G (2009). Association mapping of stigma and spikelet characteristics in rice (*Oryza sativa* L.). Mol Breed.

[CR46] Yu XQ, Mei HW, Luo LJ, Liu GL, Zou GH, Hu SP, Li MS, Wu JH (2006). Dissection of additive, epistatic and Q X E interaction of quantitative trait loci influencing stigma exsertion under water stress in rice. Acta Genet Sin.

[CR47] Yuan LP, Peng JM (2005). Hybrid rice and world food security.

[CR48] Zhou C, Liu A, Xiao C, Xie F, Hardy B (2009). Cultivation techniques for high yielding hybrid rice seed production. Accelerating hybrid rice development.

[CR49] Zhou H, Li P, Xie W, Hussain S, Li Y, Xia D, Zhao H, Sun S, Chen J, Ye H, Hou J, Zhao D, Gao G, Zhang Q, Wang G, Lian X, Xiao J, Yu S, Li X, He Y (2017). Genome-wide association analyses reveal the genetic basis of stigma exsertion in rice. Mol Plant.

