# Supplementary material for: QTL Mapping of a Novel Genomic Region Associated with High Out-Crossing Rate Derived from Oryza longistaminata and Development of New CMS Lines in Rice, O. sativa L
Source: Rice (N Y). 2021 Sep 16;14:80. doi: 10.1186/s12284-021-00521-9 (PMC8446144; doi:10.1186/s12284-021-00521-9)
Supplement: Supplementary file 1 — Additional file 1: Table S1. Correlation coefficients of the key floral traits. [file 12284_2021_521_MOESM1_ESM.pptx]

## Slide 1
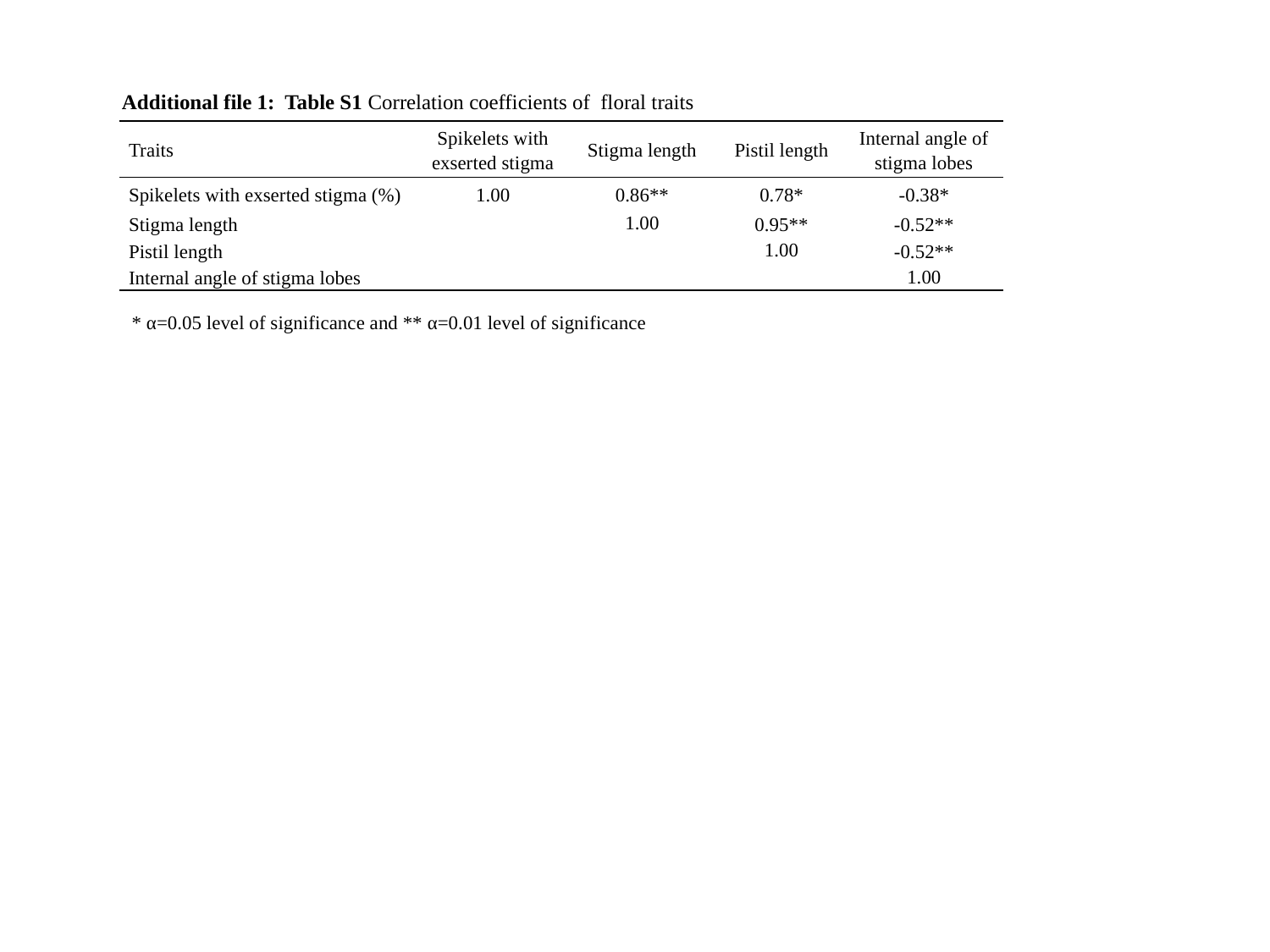

Additional file 1: Table S1 Correlation coefficients of floral traits
| Traits | Spikelets with exserted stigma | Stigma length | Pistil length | Internal angle of stigma lobes |
| --- | --- | --- | --- | --- |
| Spikelets with exserted stigma (%) | 1.00 | 0.86\*\* | 0.78\* | -0.38\* |
| Stigma length | | 1.00 | 0.95\*\* | -0.52\*\* |
| Pistil length | | | 1.00 | -0.52\*\* |
| Internal angle of stigma lobes | | | | 1.00 |
* α=0.05 level of significance and ** α=0.01 level of significance
