# Supplementary material for: QTL Mapping of a Novel Genomic Region Associated with High Out-Crossing Rate Derived from Oryza longistaminata and Development of New CMS Lines in Rice, O. sativa L
Source: Rice (N Y). 2021 Sep 16;14:80. doi: 10.1186/s12284-021-00521-9 (PMC8446144; doi:10.1186/s12284-021-00521-9)
Supplement: Supplementary file 2 — Additional file 2: Table S2. List of newly designed polymorphic InDel markers with their sequences and product sizes. [file 12284_2021_521_MOESM2_ESM.pptx]

## Slide 1
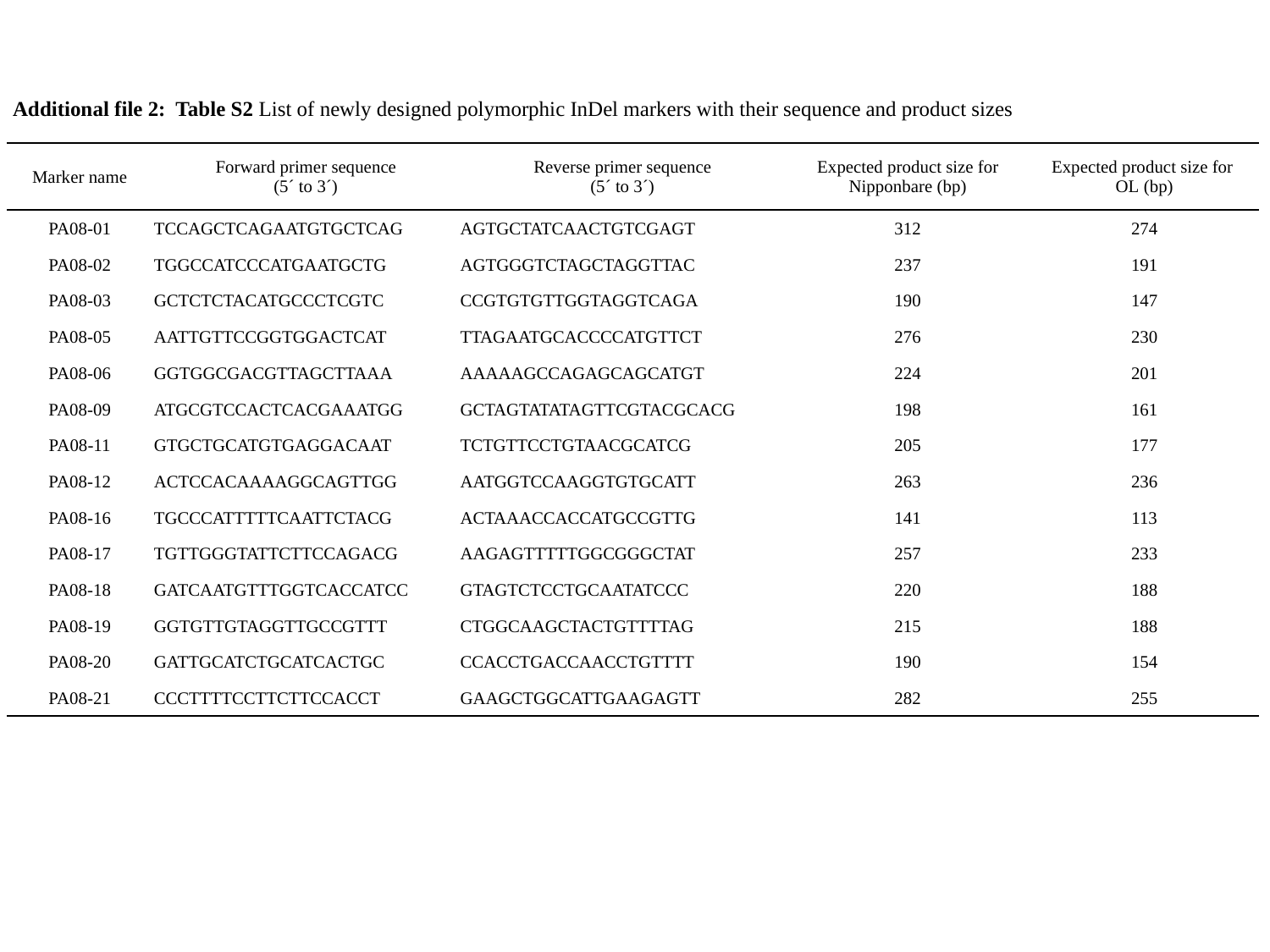

Additional file 2: Table S2 List of newly designed polymorphic InDel markers with their sequence and product sizes
| Marker name | Forward primer sequence (5´ to 3´) | Reverse primer sequence (5´ to 3´) | Expected product size for Nipponbare (bp) | Expected product size for OL (bp) |
| --- | --- | --- | --- | --- |
| PA08-01 | TCCAGCTCAGAATGTGCTCAG | AGTGCTATCAACTGTCGAGT | 312 | 274 |
| PA08-02 | TGGCCATCCCATGAATGCTG | AGTGGGTCTAGCTAGGTTAC | 237 | 191 |
| PA08-03 | GCTCTCTACATGCCCTCGTC | CCGTGTGTTGGTAGGTCAGA | 190 | 147 |
| PA08-05 | AATTGTTCCGGTGGACTCAT | TTAGAATGCACCCCATGTTCT | 276 | 230 |
| PA08-06 | GGTGGCGACGTTAGCTTAAA | AAAAAGCCAGAGCAGCATGT | 224 | 201 |
| PA08-09 | ATGCGTCCACTCACGAAATGG | GCTAGTATATAGTTCGTACGCACG | 198 | 161 |
| PA08-11 | GTGCTGCATGTGAGGACAAT | TCTGTTCCTGTAACGCATCG | 205 | 177 |
| PA08-12 | ACTCCACAAAAGGCAGTTGG | AATGGTCCAAGGTGTGCATT | 263 | 236 |
| PA08-16 | TGCCCATTTTTCAATTCTACG | ACTAAACCACCATGCCGTTG | 141 | 113 |
| PA08-17 | TGTTGGGTATTCTTCCAGACG | AAGAGTTTTTGGCGGGCTAT | 257 | 233 |
| PA08-18 | GATCAATGTTTGGTCACCATCC | GTAGTCTCCTGCAATATCCC | 220 | 188 |
| PA08-19 | GGTGTTGTAGGTTGCCGTTT | CTGGCAAGCTACTGTTTTAG | 215 | 188 |
| PA08-20 | GATTGCATCTGCATCACTGC | CCACCTGACCAACCTGTTTT | 190 | 154 |
| PA08-21 | CCCTTTTCCTTCTTCCACCT | GAAGCTGGCATTGAAGAGTT | 282 | 255 |
