# Supplementary material for: QTL Mapping of a Novel Genomic Region Associated with High Out-Crossing Rate Derived from Oryza longistaminata and Development of New CMS Lines in Rice, O. sativa L
Source: Rice (N Y). 2021 Sep 16;14:80. doi: 10.1186/s12284-021-00521-9 (PMC8446144; doi:10.1186/s12284-021-00521-9)
Supplement: Supplementary file 3 — Additional file 3: Table S3. Background genotyping analysis of the improved maintainer and CMS lines using high-resolution Infinium 7K SNP chip. [file 12284_2021_521_MOESM3_ESM.pptx]

## Slide 1
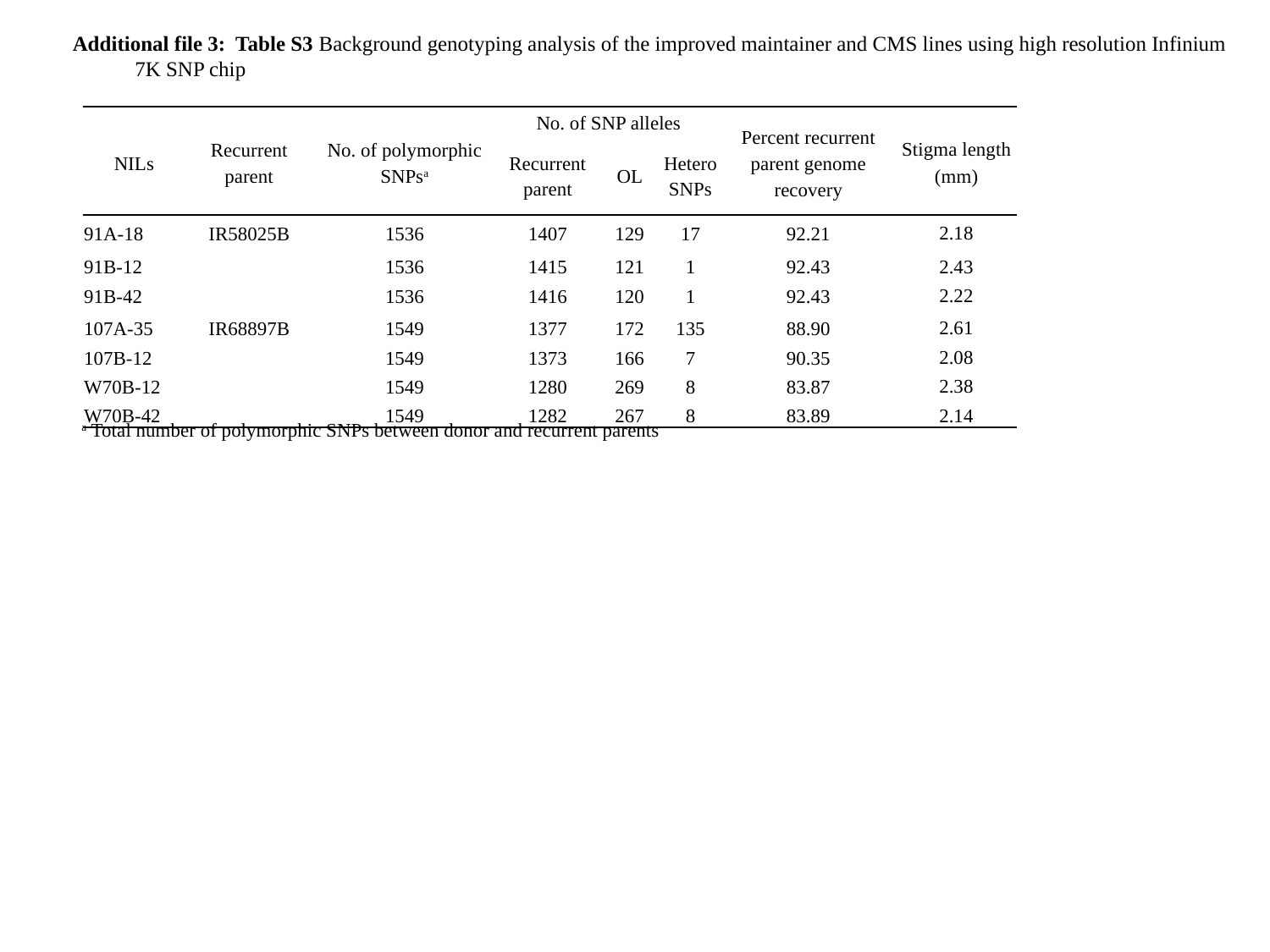

Additional file 3: Table S3 Background genotyping analysis of the improved maintainer and CMS lines using high resolution Infinium 7K SNP chip
| NILs | Recurrent parent | No. of polymorphic SNPsa | No. of SNP alleles | | | Percent recurrent parent genome recovery | Stigma length (mm) |
| --- | --- | --- | --- | --- | --- | --- | --- |
| | | | Recurrent parent | OL | Hetero SNPs | | |
| 91A-18 | IR58025B | 1536 | 1407 | 129 | 17 | 92.21 | 2.18 |
| 91B-12 | | 1536 | 1415 | 121 | 1 | 92.43 | 2.43 |
| 91B-42 | | 1536 | 1416 | 120 | 1 | 92.43 | 2.22 |
| 107A-35 | IR68897B | 1549 | 1377 | 172 | 135 | 88.90 | 2.61 |
| 107B-12 | | 1549 | 1373 | 166 | 7 | 90.35 | 2.08 |
| W70B-12 | | 1549 | 1280 | 269 | 8 | 83.87 | 2.38 |
| W70B-42 | | 1549 | 1282 | 267 | 8 | 83.89 | 2.14 |
| a Total number of polymorphic SNPs between donor and recurrent parents |
| --- |
