# Supplementary material for: QTL Mapping of a Novel Genomic Region Associated with High Out-Crossing Rate Derived from Oryza longistaminata and Development of New CMS Lines in Rice, O. sativa L
Source: Rice (N Y). 2021 Sep 16;14:80. doi: 10.1186/s12284-021-00521-9 (PMC8446144; doi:10.1186/s12284-021-00521-9)
Supplement: Supplementary file 4 — Additional file 4: Figure S1. Schematic diagram showing the different parts of the typical OL female reproductive organ (pistil). The letters a, b, c, d, e, and f denote non-brushy parts of the stigma, brushy parts of the stigma, stigma, style, ovary, and pistil, respectively. [file 12284_2021_521_MOESM4_ESM.pptx]

## Slide 1
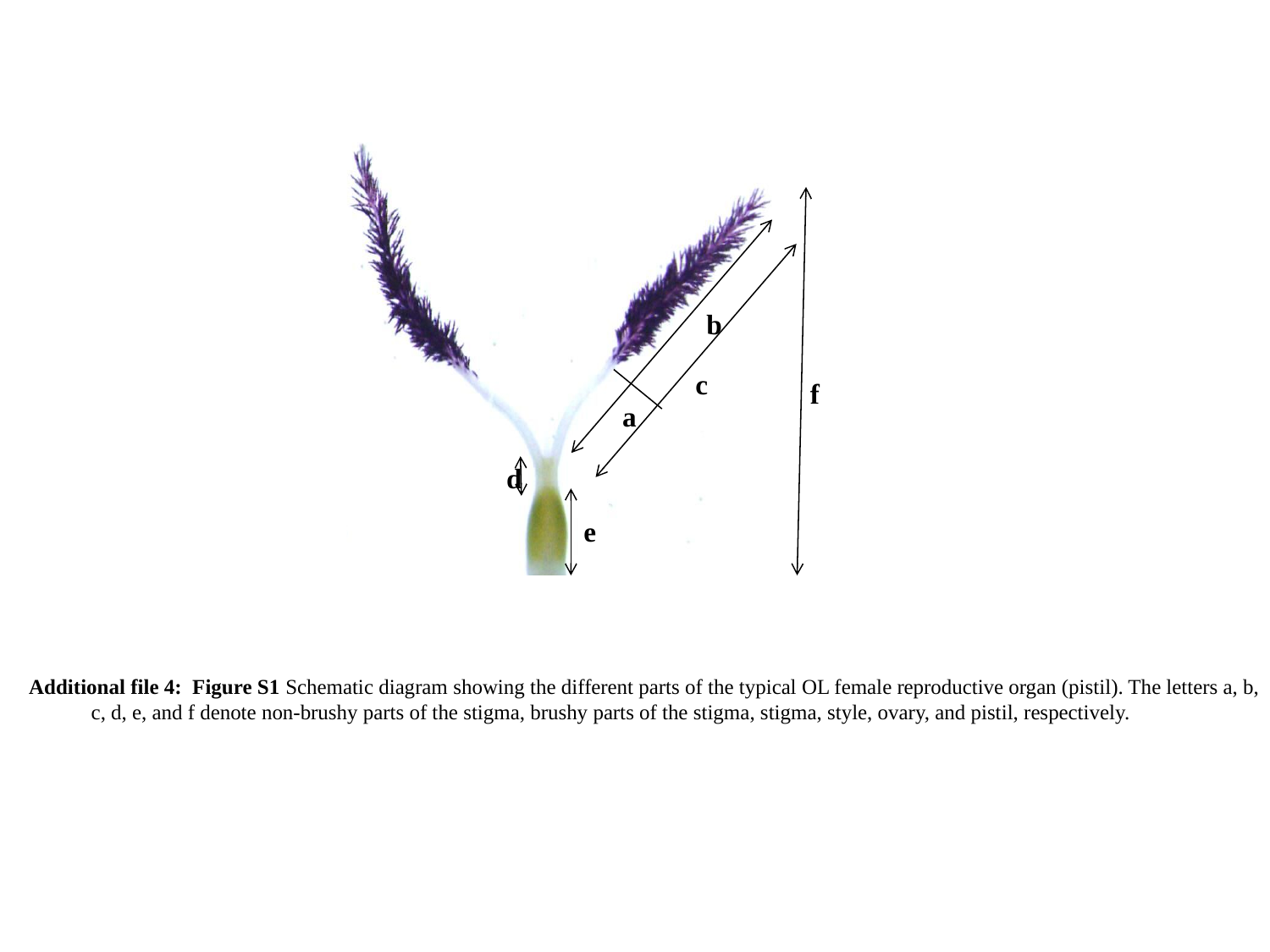

b
c
f
a
d
e
Additional file 4: Figure S1 Schematic diagram showing the different parts of the typical OL female reproductive organ (pistil). The letters a, b, c, d, e, and f denote non-brushy parts of the stigma, brushy parts of the stigma, stigma, style, ovary, and pistil, respectively.
