# Supplementary material for: QTL Mapping of a Novel Genomic Region Associated with High Out-Crossing Rate Derived from Oryza longistaminata and Development of New CMS Lines in Rice, O. sativa L
Source: Rice (N Y). 2021 Sep 16;14:80. doi: 10.1186/s12284-021-00521-9 (PMC8446144; doi:10.1186/s12284-021-00521-9)
Supplement: Supplementary file 5 — Additional file 5: Figure S2. Breeding scheme depicting the development of a mapping population derived from IR64 × OL (IRGC110404). [file 12284_2021_521_MOESM5_ESM.pptx]

## Slide 1
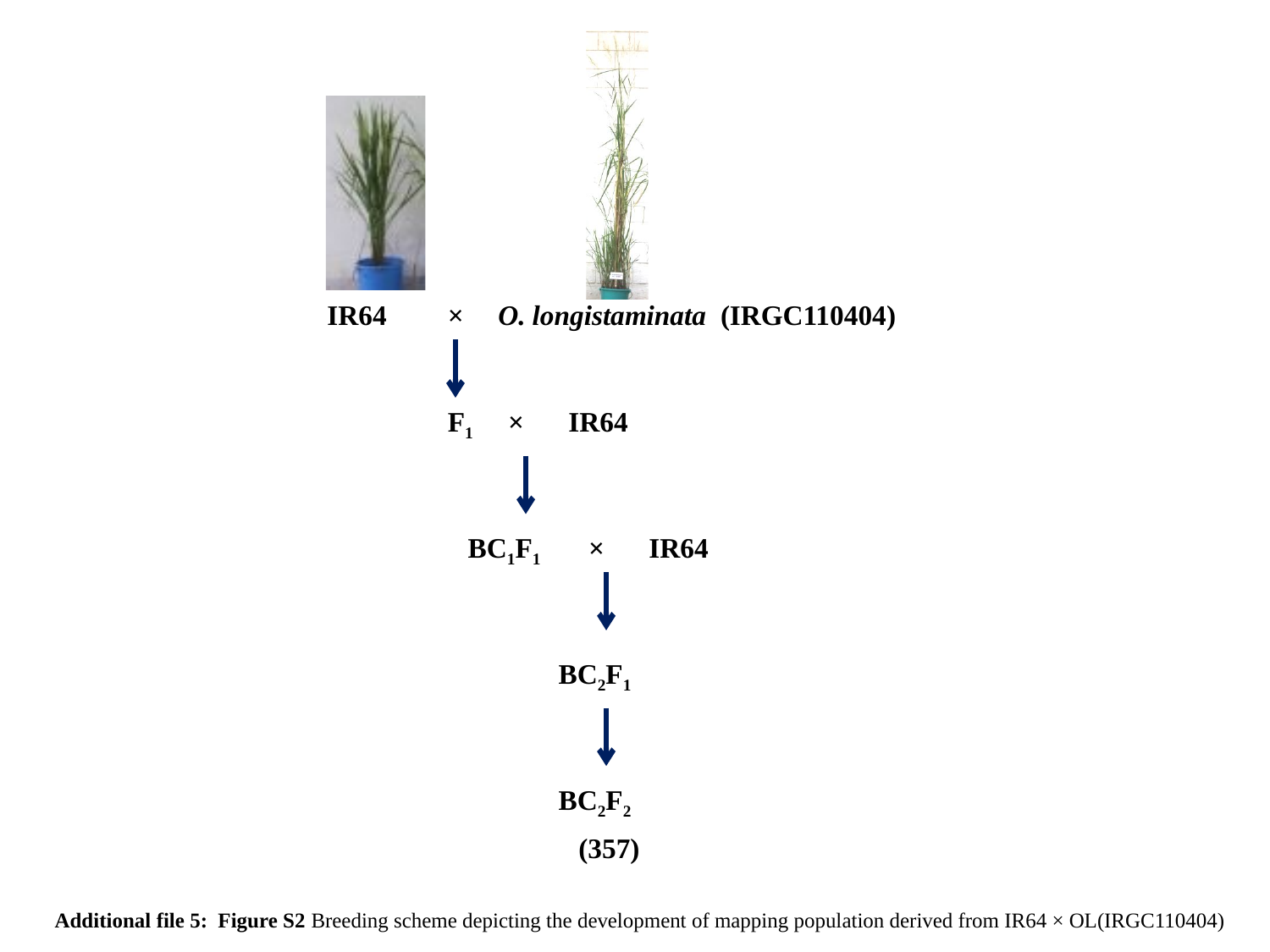

IR64
×
O. longistaminata (IRGC110404)
F1
×
IR64
BC1F1
×
IR64
BC2F1
BC2F2
(357)
Additional file 5: Figure S2 Breeding scheme depicting the development of mapping population derived from IR64 × OL(IRGC110404)
