# Supplementary material for: QTL Mapping of a Novel Genomic Region Associated with High Out-Crossing Rate Derived from Oryza longistaminata and Development of New CMS Lines in Rice, O. sativa L
Source: Rice (N Y). 2021 Sep 16;14:80. doi: 10.1186/s12284-021-00521-9 (PMC8446144; doi:10.1186/s12284-021-00521-9)
Supplement: Supplementary file 6 — Additional file 6: Figure S3. QTLs detected from 357 BC2F2 genotypes derived from an IR64 × OL cross conferring (a & b) style length, (c) stigma breadth, (d) stigma area, and (e) pistil length using 164 SSR and STS markers. [file 12284_2021_521_MOESM6_ESM.pptx]

## Slide 1
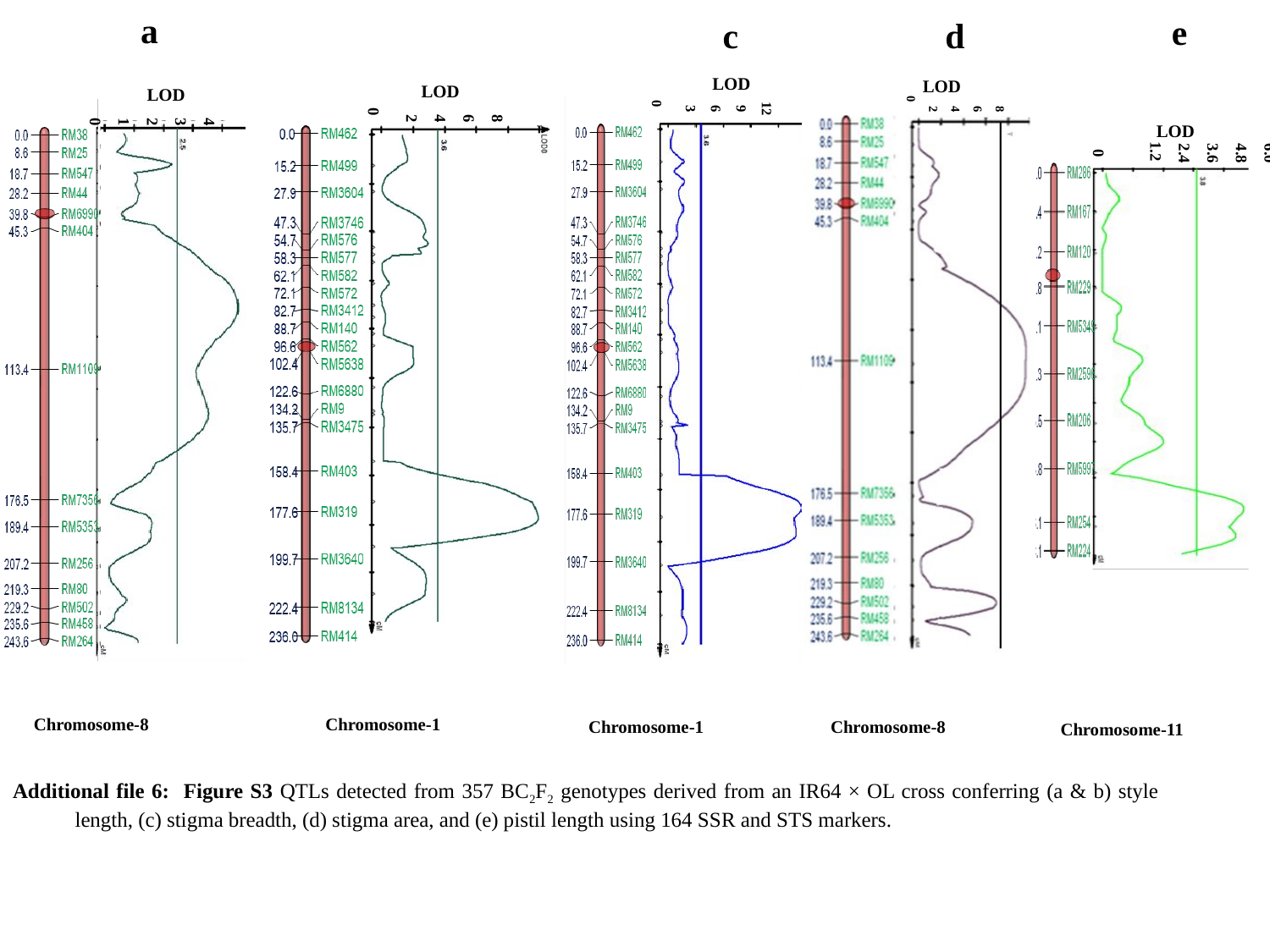

a
e
d
c
LOD
LOD
LOD
LOD
8
6
4
2
0
12
9
6
3
0
8
6
4
2
0
LOD
4
3
2
1
0
6.0
4.8
3.6
2.4
1.2
0
Chromosome-8
Chromosome-1
Chromosome-8
Chromosome-1
Chromosome-11
Additional file 6: Figure S3 QTLs detected from 357 BC2F2 genotypes derived from an IR64 × OL cross conferring (a & b) style length, (c) stigma breadth, (d) stigma area, and (e) pistil length using 164 SSR and STS markers.
