# Supplementary material for: QTL Mapping of a Novel Genomic Region Associated with High Out-Crossing Rate Derived from Oryza longistaminata and Development of New CMS Lines in Rice, O. sativa L
Source: Rice (N Y). 2021 Sep 16;14:80. doi: 10.1186/s12284-021-00521-9 (PMC8446144; doi:10.1186/s12284-021-00521-9)
Supplement: Supplementary file 7 — Additional file 7: Figure S4. Agarose (3%) gel image showing the BC2F3 co-segregation pattern of the new PA08-18 InDel marker predicted to link to qSTGL8.0. Marker alleles were scored as A for IR64 alleles, B for OL alleles, and H for heterozygous alleles of IR64 and OL for genotype score assessment. Phenotype below the genotype scores indicates length of the phenotype of the respective BC2F3 individuals. [file 12284_2021_521_MOESM7_ESM.pptx]

## Slide 1
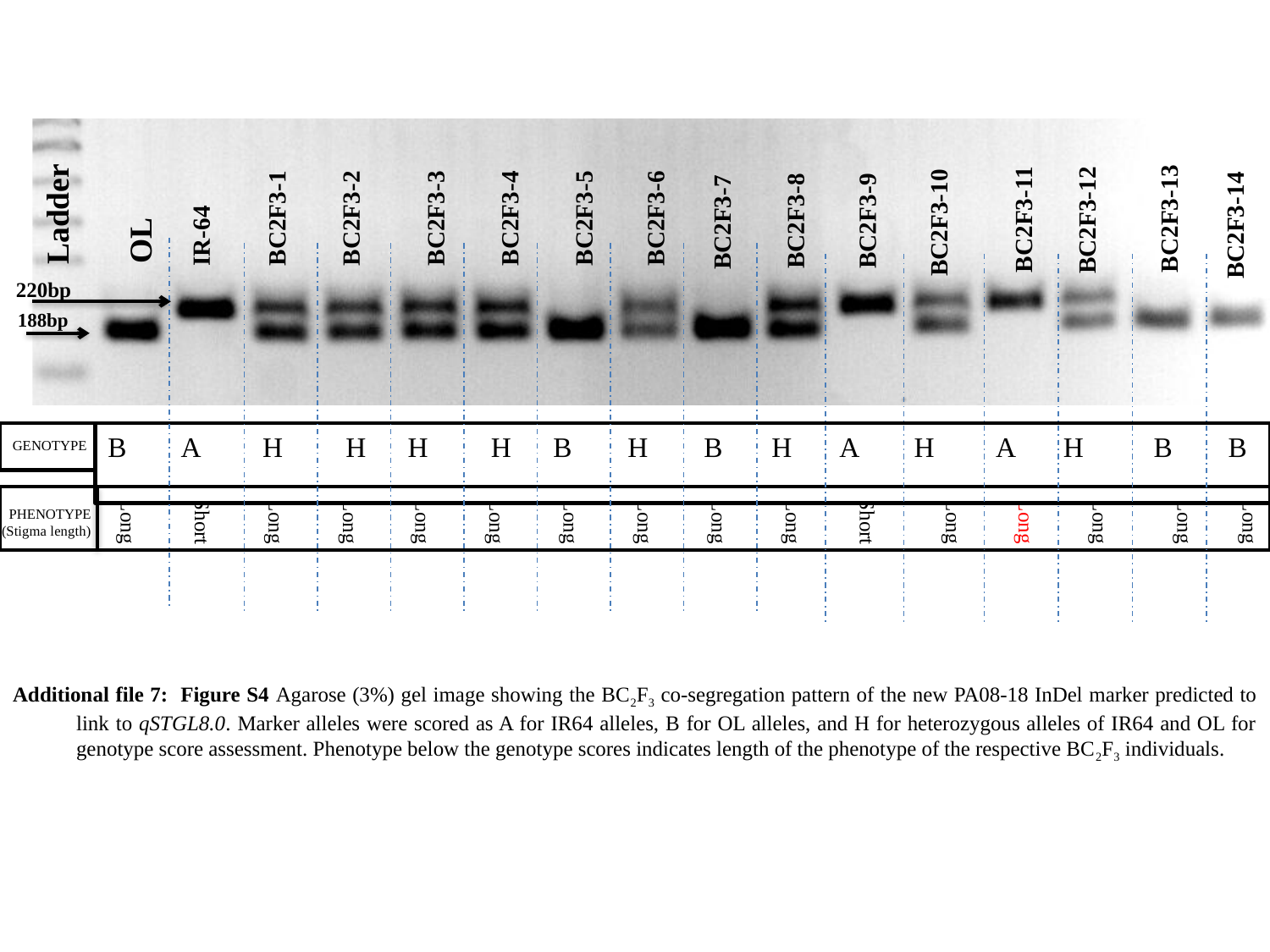

OL
Ladder
BC2F3-1
BC2F3-2
BC2F3-3
BC2F3-4
BC2F3-5
BC2F3-6
BC2F3-8
BC2F3-9
BC2F3-7
BC2F3-11
BC2F3-12
BC2F3-10
BC2F3-14
BC2F3-13
IR-64
220bp
188bp
B A H H H H B H B H A H A H B B
GENOTYPE
Long
Short
Long
Long
Long
Long
Long
Long
Long
Long
Short
Long
Long
Long
Long
Long
 PHENOTYPE
(Stigma length)
Additional file 7: Figure S4 Agarose (3%) gel image showing the BC2F3 co-segregation pattern of the new PA08-18 InDel marker predicted to link to qSTGL8.0. Marker alleles were scored as A for IR64 alleles, B for OL alleles, and H for heterozygous alleles of IR64 and OL for genotype score assessment. Phenotype below the genotype scores indicates length of the phenotype of the respective BC2F3 individuals.
