# Supplementary material for: QTL Mapping of a Novel Genomic Region Associated with High Out-Crossing Rate Derived from Oryza longistaminata and Development of New CMS Lines in Rice, O. sativa L
Source: Rice (N Y). 2021 Sep 16;14:80. doi: 10.1186/s12284-021-00521-9 (PMC8446144; doi:10.1186/s12284-021-00521-9)
Supplement: Supplementary file 8 — Additional file 8: Figure S5. Schematic diagram showing the breeding scheme of an IR68897B ×OL(IRGC92664) cross for the development of improved maintainer (a) and CMS lines (b) with long-exserted stigma and higher out-crossing rate than recurrent parents IR68897B and IR68897A, respectively. The same breeding scheme was used for an IR58025B ×OL (IRGC110404) cross to develop long-exserted stigma lines in IR58025B and IR58025A backgrounds. [file 12284_2021_521_MOESM8_ESM.pptx]

## Slide 1
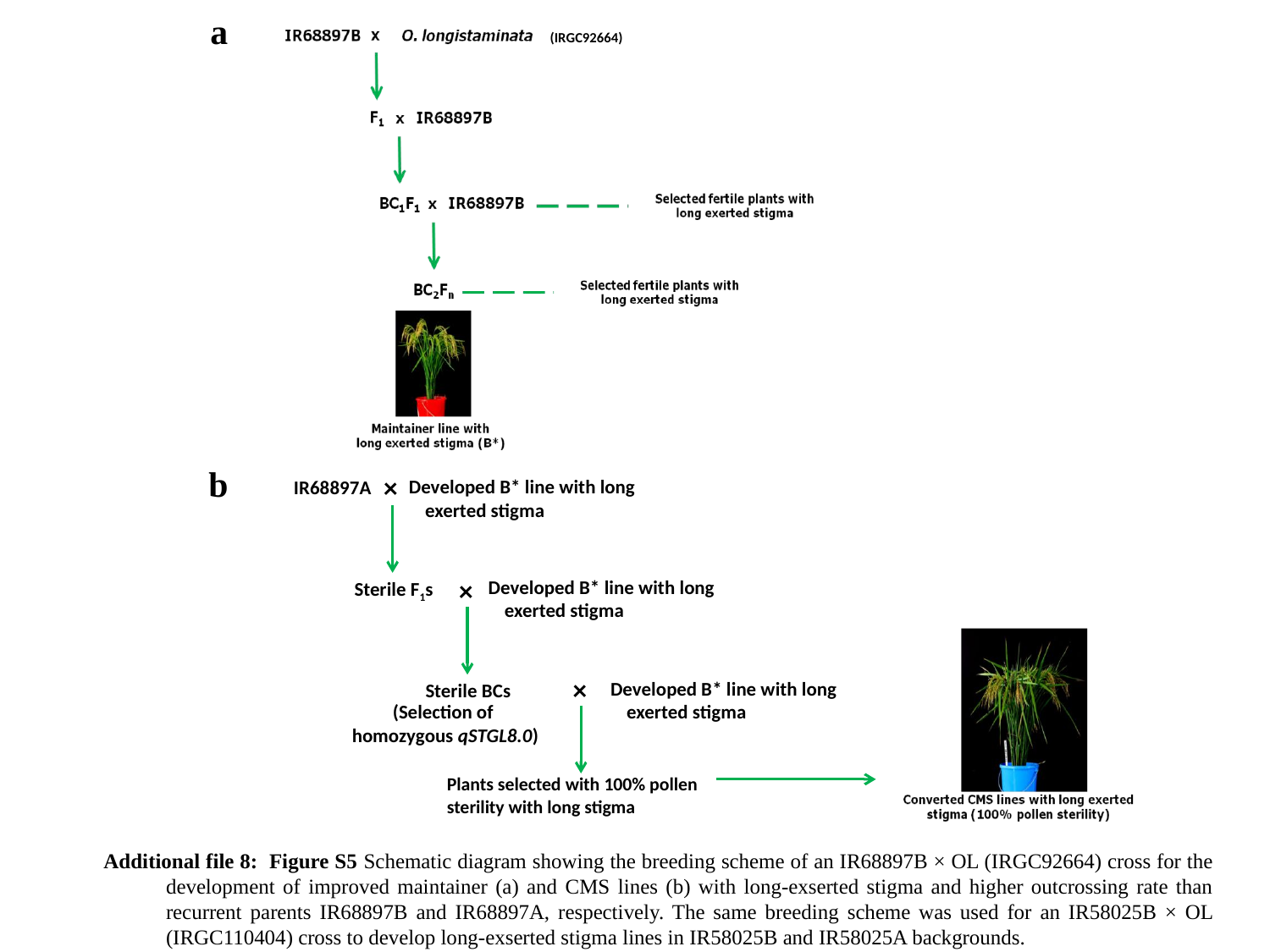

a
(IRGC92664)
b
×
IR68897A
Developed B* line with long exerted stigma
×
Developed B* line with long exerted stigma
Sterile F1s
×
Developed B* line with long exerted stigma
Sterile BCs
(Selection of
homozygous qSTGL8.0)
Plants selected with 100% pollen sterility with long stigma
Additional file 8: Figure S5 Schematic diagram showing the breeding scheme of an IR68897B × OL (IRGC92664) cross for the development of improved maintainer (a) and CMS lines (b) with long-exserted stigma and higher outcrossing rate than recurrent parents IR68897B and IR68897A, respectively. The same breeding scheme was used for an IR58025B × OL (IRGC110404) cross to develop long-exserted stigma lines in IR58025B and IR58025A backgrounds.
