# Supplementary material for: QTL Mapping of a Novel Genomic Region Associated with High Out-Crossing Rate Derived from Oryza longistaminata and Development of New CMS Lines in Rice, O. sativa L
Source: Rice (N Y). 2021 Sep 16;14:80. doi: 10.1186/s12284-021-00521-9 (PMC8446144; doi:10.1186/s12284-021-00521-9)
Supplement: Supplementary file 9 — Additional file 9: Figure S6. Phenotypes of panicle, floret, and stigma. (a) IR68897B. (b) OL (IRGC110404). (c) The improved IR58025B possessing qSTGL8.0 (IRGC110404). (d) The improved IR68897B possessing qSTGL8.0 (IRGC92664). Red arrows point to the exserted stigma in each genotype. [file 12284_2021_521_MOESM9_ESM.pptx]

## Slide 1
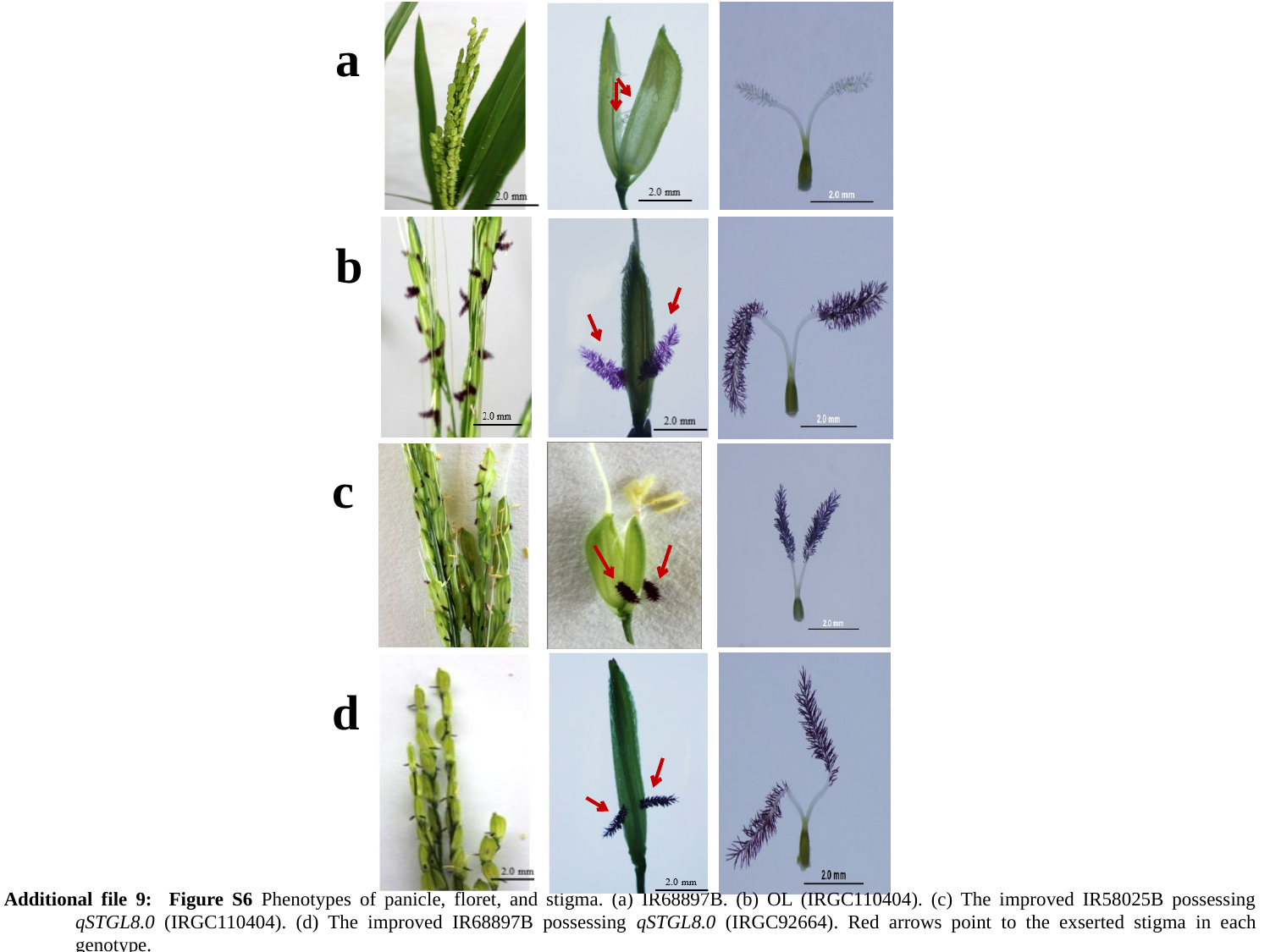

a
b
c
d
Additional file 9: Figure S6 Phenotypes of panicle, floret, and stigma. (a) IR68897B. (b) OL (IRGC110404). (c) The improved IR58025B possessing qSTGL8.0 (IRGC110404). (d) The improved IR68897B possessing qSTGL8.0 (IRGC92664). Red arrows point to the exserted stigma in each genotype.
